# International Overview of Somatic Dysfunction Assessment and Treatment in Osteopathic Research: A Scoping Review

**DOI:** 10.3390/healthcare10010028

**Published:** 2021-12-24

**Authors:** Marco Tramontano, Federica Tamburella, Fulvio Dal Farra, Andrea Bergna, Christian Lunghi, Mattia Innocenti, Fabio Cavera, Federica Savini, Vincenzo Manzo, Giandomenico D’Alessandro

**Affiliations:** 1 Fondazione Santa Lucia IRCCS, 00179 Roma, Italy; f.tamburella@hsantalucia.it; 2 Research Department, SOMA Istituto Osteopatia Milano, 20126 Milan, Italy; fulviodalfarra@outlook.it (F.D.F.); andreabergna@soma-osteopatia.it (A.B.); 3 AISO-Associazione Italiana Scuole di Osteopatia, 65125 Pescara, Italy; 4 Clinical-Based Human Research Department, Research Division, Foundation COME Collaboration, 65121 Pescara, Italy; christianlunghi@gmail.com (C.L.); giandosteo89@hotmail.it (G.D.); 5 Centre pour l’Etude, la Recherche et la Diffusion Ostéopathiques “C.E.R.D.O.”, 00199 Rome, Italy; mattiainnocenti6@gmail.com (M.I.); Fabiocavera4@gmail.com (F.C.); fe.savini@gmail.com (F.S.); direzione@cerdo.it (V.M.)

**Keywords:** osteopathic manipulative treatment, somatic dysfunction, manual therapy, osteopathic techniques

## Abstract

Background: Osteopathic manipulative treatment (OMT) is a patient-centred, whole-body intervention aimed at enhance the person’s self-regulation. OMT interventions are focused on somatic dysfunctions (SD) that can be defined as an altered regulative function associated with inflammatory signs palpable in the body framework in different body regions. The conceptual model that sustains SD, as well as its usefulness for the osteopathic profession, is still being discussed by the osteopathic community. Understanding the role and the application of SD is the aim of this scoping review. Methods: A literature search was carried out through the main biomedical databases: Pubmed (Medline), Cochrane, Central (Cochrane), Embase, PEDro and Scopus. Grey literature was considered via Google Scholar and the Osteopathic Research Web. The review was prepared by referring to the “Preferred Reporting Items for Systematic reviews and Meta-Analysis extension for Scoping Reviews” (PRISMA-ScR). Results: A total of 37,279 records were identified through database searching and other sources. After the duplicates were removed, 27,023 titles and abstracts were screened. A total of 1495 full-text articles were assessed for eligibility. The qualitative synthesis included 280 studies. Conclusions: Treating SD is an important part of osteopathic practice that varies from country to country. SD should be considered as a clinical value that assists in the clinical assessment and guides the decision-making process of osteopathic practitioners. Further studies should be designed to better understand why and how to choose the different assessment and intervention modalities to approach SD and to evaluate new osteopathic models.

## 1. Introduction

Osteopathic manipulative treatment (OMT) is a patient-centred, whole-body intervention. It is aimed at enhancing the person’s self-regulation and favor the structure-function-environment relationship by applying five models as a guide for the osteopathic approach to diagnosis and treatment [1,2]. Osteopathy is recognised and regulated differently throughout the world [3]. In fact, in the USA there is an ongoing process to establish osteopathic principles to fulfill osteopathic recognition standards [4]; many countries, for example, Italy [5], are struggling to spread a shared benchmark for standardized core curriculum and core competence. In many European countries, efforts are being made to have osteopathy confirmed as a healthcare profession by identifying distinctive osteopathic competencies and producing evidence. As recently reported in a bibliometric analysis, osteopathic research has increased rapidly in recent years [6]. The main advantage for patients undergoing OMT is the effective relief of acute and chronic pain [7,8]; however, patients also report improvement of other non-musculoskeletal complaints [9,10]. Indeed, some of the latter are conditions and disorders that are closely associated with other neurological functions, including reduced time of hospitalization in a large population of preterm infants [11,12] and autonomic and neuroendocrine responses [13]. The mechanisms of action behind osteopathic interventions are being debated; however, recent studies have reported that one possible explanation of OMT effectiveness is that it modulates neurophysiological and cerebral activities [14,15,16]. Indeed, cerebral perfusion and heart rate variability changes were recently found in patients with low back pain after OMT [17]. Osteopaths often use many different techniques in the same session and different regions of the body are targeted by the intervention [6]. Since OMT techniques can have different effects on neurophysiological activities [18,19,20] their choice during treatment is a clinical judgment of the practitioner. OMT person-centred interventions should focus on somatic dysfunctions (SD) [21,22,23] detected in shared decision-making [24]. SD can be defined as an altered regulative function associated with inflammatory signs palpable in the body framework in different body regions that can be remote from the symptomatic area [25]. Although the clinical application of five models (biomechanical, respiratory/circulatory, neurological, biopsychosocial and bioenergetic) could provide a framework for interpreting the significance of a SD [21], thus guiding the osteopath through assessment and treatment, how osteopaths manage the decision-making process and why they choose one type of treatment over another is still unclear. Indeed, treatments can be performed following different paradigms by considering a whole-body assessment and treatment (i.e., black-box protocol) or only one or some regions of the body in a standardised or semi-standardised protocol. In any case, SD is considered to be a profession-specific approach because it defines the aim of an osteopathic evaluation and treatment (i.e., to improve a regulative function with a tailored touch delivered on an area of interest for patient and practitioner).

On the other hand, SD is also a potentially helpful concept for sharing the object of the osteopathic person-centred intervention with mainstream healthcare professionals because it is described in the International Classification of Diseases (ICD), which reports the different locations of body regions [21]. Nevertheless, the conceptual model that sustains SD, as well as its usefulness for the osteopathic profession, is still being discussed by the osteopathic community [26,27,28,29,30,31,32]. Understanding the role of SD is essential for many of those involved in the healthcare and education system [33]. To date, no reviews have systematically analysed the use of SD for assessment and treatment in osteopathic clinical practice. This scoping review was aimed at mapping the scientific literature that clarifies the use, role and application of SD in the osteopathic field by reporting details regarding physical examinations, modalities and time frame of treatment and the characteristics of the profession.

## 2. Materials and Methods

### Registration and Reporting

The current scoping review was prepared by referring to the “2020 version of the Joanna Briggs Institute Reviewers’ Manual” [34] and to the “Preferred Reporting Items for Systematic reviews and Meta-Analysis extension for Scoping Reviews” (PRISMA-ScR) [35]. The protocol was designed by three osteopaths with more than 10 years of experience in both clinical and scientific fields and was registered with protocol number 10.17605/OSF.IO/VZGPU on Open Science Framework Registry.

This review was carried out in 5 steps: (1) identifying the research question; (2) identifying relevant studies; (3) study selection; (4) charting the data; and (5) collating, summarising and reporting the results.

Step 1: Identifying the research question

The aim of this paper was to map the existing literature on the role and use of SD in osteopathic research. For this purpose, we formulated the following two research questions: “What can be gleaned from the current literature about the use of SD in osteopathic research?” “How SD is eventually considered and approached in osteopathic trials?”.

Step 2: Identifying relevant studies

The literature search was carried out through the main biomedical databases, i.e., Pubmed (Medline), Cochrane, Central (Cochrane), Embase, PEDro and Scopus. Grey literature was considered via Google Scholar and the Osteopathic Research Web. So as not to miss any relevant sources, additional records were searched through cross-referencing. The search was conducted up until July 2021, with monthly updates.

Search strategy

For Ovid MEDLINE, the initial search strategy was created using medical subject headings (MeSH) and text words (Table S1). This was then tweaked to fit the syntax and subject headings of the other databases that were searched.

Eligibility Criteria: inclusion and exclusion criteria

Studies were included when they met specific criteria in terms of population, concept and context; we considered works dealing with any type of participant, i.e., patients (male or female) asymptomatic and affected by all the conditions generally treated by osteopathic manipulation (i.e., musculoskeletal complaints, neurological, orthopaedic, pediatric, gastroenteric) have been accepted. As for the concept, any study that focused on a specific osteopathic manipulation was included in each possible context. The osteopathic approaches considered were myofascial release (MFR), high-velocity low-amplitude (HVLA) and muscle energy techniques (MET), cranio-sacral treatment (CST), visceral manipulation (VM) and two or more of these combined in a standardised, semi-standardised or black-box modality. Any study design or type of publication that concerned the effects of the osteopathic treatment was accepted without any restrictions in terms of time, setting, country or language. We excluded studies that considered a single osteopathic technique or that did not clearly specify a particular osteopathic approach (e.g., manual therapy, chiropractic therapy).

Step 3: Study selection

**Figure 1:**
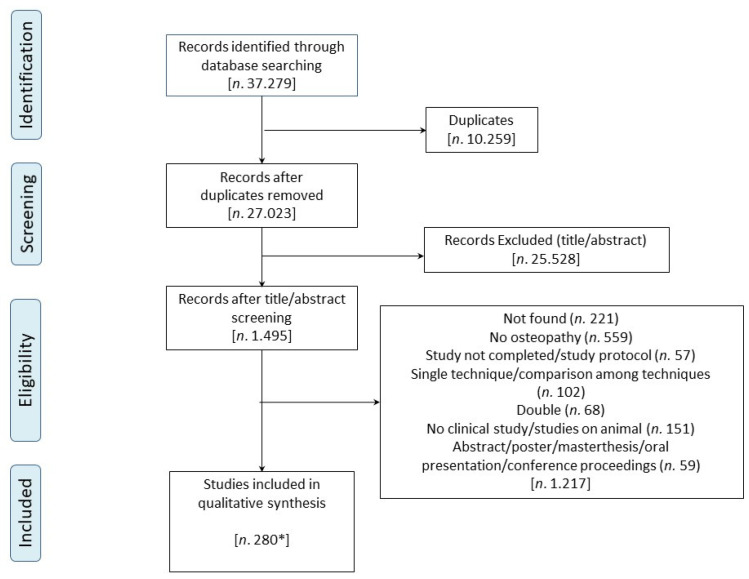
PRISMA flow diagram. * Two articles were considered doubled.

The search strategy results were uploaded and managed through Zotero [36] i.e., online software of the Corporation for Digital Scholarship, which was developed by a global community for collecting, organising, citing and sharing research. Duplicates were automatically removed; records were screened first by title and abstract and second by full-text reading. Both phases were conducted by starting with a pre-screening team meeting to discuss inclusion and exclusion criteria. To test the consistency of extracting and reporting methods, 24 h of training were carried out by the reviewers in five online meetings. The reviewers independently screened the articles; disagreements were resolved through discussion and consensus. Further details of this review process are better illustrated in the PRISMA flow diagram (Figure 1).

Step 4: Charting the data

The data extraction form used to collect the data had been developed previously and was discussed, implemented and accepted by all authors of the study. The following information was included: year of publication and country, type of journal, study aim, study design and duration, sample size and characteristics, description of the intervention, details concerning somatic dysfunction, osteopathic approach and outcomes considered.

Step 5: Collating, summarising and reporting the results

Data were reported numerically and thematically. Descriptive statistics were used to report means, standard deviations, median, interquartile ranges and percentages for all the considered outcomes, such as period of publication, country, study design, characteristics of participants, assessment and interventions. Extracted data were reported by considering the role and use of SD: (1) in the assessment; (2) in the treatment and (3) in the time frame of treatment. More specifically, a descriptive summary that details the overall number of studies included in the review, their characteristics as well as the data extracted from the included studies, was provided. A list of full texts is reported in Table S2. To consider a large number of variables, we opted to use several graphs to facilitate the reading and interpretation of the results. PRISMA-ScR Checklist is reported in Table S3.

## 3. Results

### 3.1. Study Selection

A total of 37,279 records were identified through database searching and other sources. After the duplicates were removed, 27,023 titles and abstracts were screened. Then, 1495 full-text articles were assessed for eligibility and 1217 articles were excluded because they did not fulfill the inclusion criteria or because full texts were unavailable. The qualitative synthesis included 278 articles. Two articles were considered doubled because there was a specific description of the two case reports. Therefore, 280 studies were included in this scoping review. 

### 3.2. Characteristics of the Included Studies

**Figure 2:**
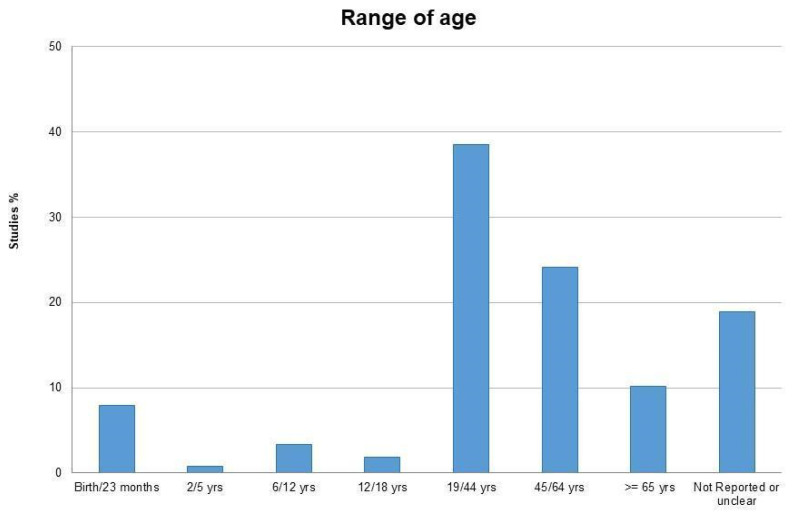
Classification by age categories of individuals enrolled in the studies included in this review, reported as percentages with respect to the sample totality.

Overall, 280 studies were included for a total of 16,731 participants; 6792 were females (40.6%). Participants’ mean age was 38.1 ± 21.93 years (details are reported in Figure 2). 86.3% of the included studies were carried out on symptomatic participants. Of these, 166 were clinical trials, both randomized and non-randomized, 80 were case reports and 39 included case series, cohort and case-control studies. OMT was performed by professional osteopaths in 156 studies (55.7%), by osteopathy students in 19 studies (6.7%) and by “others” (e.g., physicians, pediatricians or physiotherapists) in 15 studies (5.3%).

33.3% of the studies reported unclear or no information about the researcher who performed OMT.

### 3.3. Data Synthesis

#### 3.3.1. Somatic Dysfunction in the Assessment

**Figure 3:**
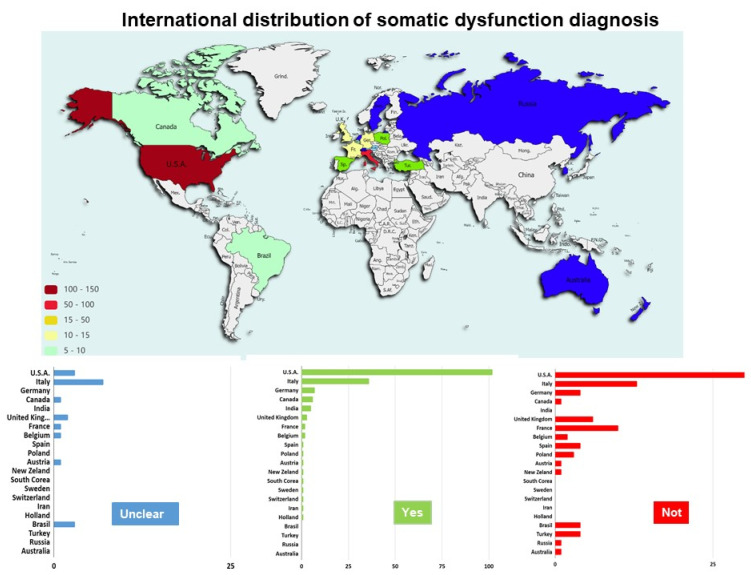
International distribution of somatic dysfunction diagnosis, reported as the number of studies investigating (Yes) or not (Not) the somatic dysfunction for each country. Studies for which it was not clear the diagnosis of somatic dysfunction were reported (Unclear).

The percentage of SD used in the different studies varied from country to country (Figure 3); some countries (e.g., the USA, Italy and India) considered SD more than others (e.g., Germany, France, UK and Brazil).

SD was considered in 170 studies; 95 studies did not report any information about the use of SD; in 20 studies, this information was unclear. In 10 studies, ICD was considered to report SD.

In 214 studies, the tenderness, asymmetry, restricted motion, and tissue texture (TART criteria) was not used to identify SD; only 15.8% of the included studies adopted it; 4.2% used other methods; finally, in 4.9% there was unclear information about the parameters used to find SD.

Six studies reported SD through the subjective, objective, assessment, and plan (SOAP note); one study used another method; 97.65% used no form.

SDs were considered locoregional in 56 studies (20%), and segmental in 53 studies (18.9%), both locoregional and segmental in 6 studies (2.1%); in 55 studies (19.6%) there was unclear information about this aspect.

In 155 studies, practitioners used an SD-based treatment. However, the authors of 73 of these studies did not clearly state why they used it. In 105 studies, professional osteopaths based treatment on SD assessment; in 26 studies SD was not considered; in 25 studies methods were unclear about this procedure; SD was primarily adopted by osteopathic students in 8 out of 19 studies; in 3 studies SD was not considered; in 8 studies methods about the SD-based treatment were unclear. As to the “other” category, which included physicians and physiotherapists, SD-based treatments were carried out in 5 studies, 4 studies did not use them, and 4 studies were methodologically unclear.

#### 3.3.2. Somatic Dysfunction in the Treatment

**Figure 4:**
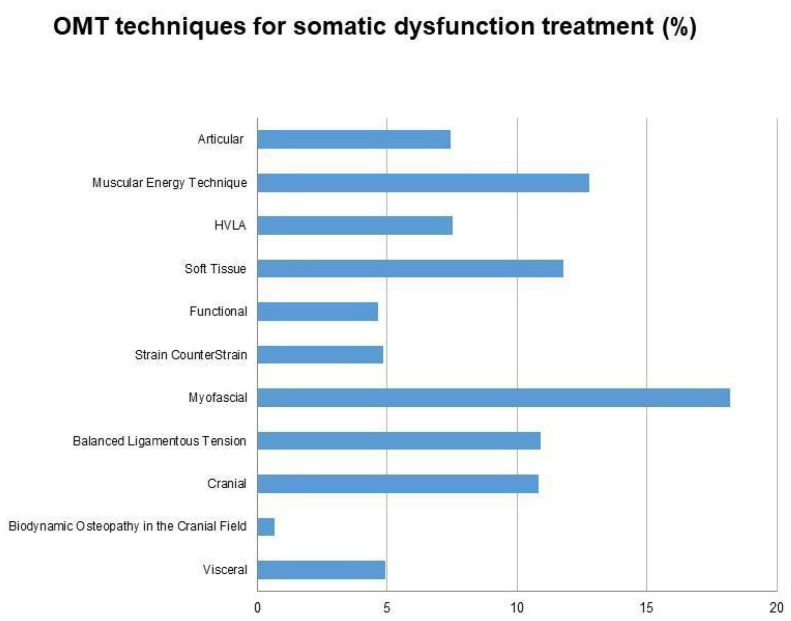
OMT techniques used for the treatment of somatic dysfunction across the studies included in the review. OMT = Osteopathic Manipulative Treatment.

Different techniques were adopted in the included studies. The most common approaches were myofascial (77.8%), muscle energy technique (55.6%), soft tissue (50.8%), balanced ligamentous tension and cranial approaches (46.6%). The less common approaches were strain and counterstrain and visceral (20.8%), and biodynamic osteopathy in the cranial field/fluidic techniques (2.6%). 91.0% of the studies used two or more techniques, while 9.0% used a single technique (details of the osteopathic techniques used are reported in Figure 4).

Treatment modality was reported in 273 studies and was classified as black-box (if a whole-body assessment and treatment was considered), standardised (if the same regions were always treated in a protocol order) and semi-standardised (if one or more regions of the body were treated). A total 37.5% of the included studies used the black-box methodology for treatment, 33.3% a standardised treatment and 24.9% a semi-standardised modality.

**Figure 5:**
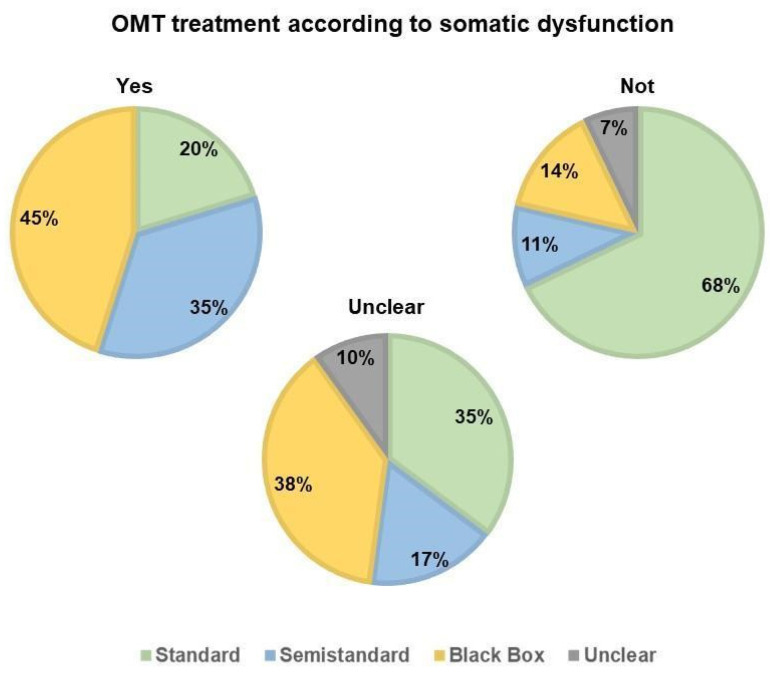
OMT treatment (standard, semistandard, black box or unclear) according to somatic dysfunction across the studies included in the review. Yes: OMT treatment was based on somatic dysfunction; Not: OMT treatment was not based on somatic dysfunction; Unclear: it was not defined if OMT treatment was based on somatic dysfunction. OMT = Osteopathic Manipulative Treatment.

A total 4.2% reported insufficient information about the treatment. Thus, it was impossible to determine whether standardised, semi-standardised or black-box treatment was performed. Figure 5 summarises the percentage of treatment modalities used related to the SD; 90.5% reported no information about the occurrence of side effects; 18 studies reported no side effects; 5 reported low side effects; 3 reported moderate side effects and 1 reported severe side effects.

#### 3.3.3. Somatic Dysfunction in the Time Frame of Treatment

The effectiveness of one or more sessions of OMT was evaluated in 156 studies; 66 articles evaluated the effects of a single session of OMT; 101 used 2 to 5 OMT sessions; in 79 studies more than 6 sessions were carried out. In the remaining 39 studies, unclear information was reported regarding the number of treatment sessions used. Study duration, i.e., from enrollment to follow-up, varied among studies: 110 studies (39.2%) lasted 3 months or less, 69 studies (24.6%) lasted from 7 months to 1 year; 58 studies (20.7%) had a duration of 13 months or more; 67 studies (23.9%) did not report any information about study duration.

In the studies that included more than one treatment, the frequency of OMT was 1 treatment/week in 62 studies; in 57 studies it was less than 1 treatment/week (e.g., 3 treatments per month), 2 treatments/week in 31 studies, ≥4 treatments/week in 19 studies and 49 studies did not report any information about the frequency of the sessions or reported unclear information.

Regarding session duration, in 61 studies the OMT session lasted less than 30 min; in 97 studies it lasted 30 or more min; 87 studies did not report information about the duration of the OMT sessions and 43 studies reported unclear information about it.

155 studies (55.3%) had a follow-up control and the other 130 did not.

## 4. Discussion

This study, which mapped the scientific literature discussing the role of SD in osteopathic field, includes information about assessment, modalities and time frame of treatment and professional characteristics. Based on the 280 studies included, we were able to identify heterogeneous roles and applications of SD. This empirical evidence of heterogeneity highlights the need for an international consensus within the osteopathic practice community regarding a reliable and valid standardised assessment method that justifies the proposed manual treatment, to make the research more homogeneous and to allow for the easier synthesis of findings and outcomes.

Despite a shared set of core competencies reported by the Osteopathic International Alliance, it included detecting SD for the osteopathic diagnosis [37]. The results of the present review highlight the fact that the consideration of SD in osteopathic practice is not the same throughout the world. For example, in the USA there is an explicit mention of SD in the Core Competencies document prepared by the American Association of Colleges of Osteopathic Medicine, in conjunction with all U.S. Osteopathic Medical Schools [38]. In Europe, primarily because of the different stages of recognition and regulation processes in the different countries, SD is not always mentioned in the requested standard of practice of the professions. For example, in Italy, osteopathy has recently been recognised by law and regulated as a health profession able to manage health conditions that require interventions for prevention and maintenance of health through osteopathic treatment of SDs [39]. Conversely, there is no mention of SD in the U.K.’s osteopathic practice standard [40]. In the presented report, the studies in which authors implemented a validated system for recording, collecting and evaluating clinical findings, SDs are clearly labelled. The American Academy of Osteopathy’s Louisa Burns Osteopathic Research Committee designed, published, distributed and highly recommended the SOAP to the osteopathic profession for research, training, and clinical practice [41]. It is an easy-to-use, validated system for recording, collecting and evaluating clinical findings, osteopathic musculoskeletal examinations, enumerating any SDs found, documenting any osteopathic techniques used and reporting patient response to treatment [41]. Despite this, the SOAP note is hardly used in the selected studies. Similarly, most of the studies do not use the clinical signs identified to date to detect SD, i.e., those represented by the acronym TART: tenderness, asymmetry, restricted motion, tissue texture. In addition, one of the most widely used and recognised international disease classification systems, the ICD, which identifies SD as a “biomechanical lesion not elsewhere classified” by identifying the relevant body regions where it might be located (skeletal regions), is not yet used by the osteopathic practice community to align with other healthcare professions with respect to clinical classification. The non-homogeneity of the osteopathic evaluation, taking into account the SD collected in this review, highlights the need to use other methods that clarify the clinical signs collected during the osteopathic physical examination, to make them more reliable, valid, understandable by other healthcare professions and accessible during osteopathic education. For this reason, some osteopaths have begun to propose and study new models revisiting the clinical signs that are useful for detecting SD, while maintaining the real context of osteopathic clinical practice. For example, Bergna et al. [32] suggested “motion variability” as a clinical sign to be considered in the objective examination characterising osteopathic practice that is useful for clinical reasoning and directing OMT. The definition of SD refers to an altered regulative function associated with inflammatory signs palpable in the body framework [42]. Rather than a disease, it is regarded as a factor that contributes to and maintains patient symptoms [7]. In recent years, osteopathic researchers have referred to SD as a neurologically active area, region or a generalised body pattern to be used by osteopaths to deliver the effects of touch and other hands-off procedures to improve the patient’s agency [24]. Nevertheless, there is still a trend to use the term “osteopathic lesion” inside the osteopathic community, illustrating the adoption and misappropriation of a biomedical word and the potential nocebo effect on symptoms [33].

Concerning the different considerations of the concept of SD found in the included studies, it should be mentioned that, in 2020, authors from outside the U.S., i.e., the European, Australasian and Brazilian communities of practice, published their contributions to a debate about osteopathic conceptual models, including the concept of SD [26,27,28,29,30,42,43,44,45,46]. On one hand, Esteves et al. mentioned SD as a non-relevant clinical entity [26]; on the other hand, different authors discussed updating the concept and renovating the old theoretical substrate, not necessarily removing it [27,29,30,42,43,44,45,46,47]. They claimed that it is possible to gain a conceptual osteopathic approach that uses SD as a reconditioned model by implementing new evidence-based knowledge, such as allostasis and interoception, involving (en) active model and strategies, integrating patient-centred communication, shared decision-making self-management, and educational coaching [27]. All authors agreed that the time has arrived to build teamwork among global osteopathic communities to produce a shared vision and reduce the gap between scientific knowledge and osteopathic tradition. There is an opportunity to move forward from pseudo-scientific concepts and adopt a person-centred and evidence-informed osteopathic practice [26,27,47,48].

Regarding intervention, the results show the heterogeneity of the osteopathic techniques reported in the included studies regarding intervention. Across included studies, the OMT techniques selected by practitioners highlighted a preponderance of soft tissue techniques, such as myofascial and muscular energy techniques. On the contrary, very few studies reported the choice of biodynamic osteopathy in the cranial field. All of the main osteopathic approaches reported in the Glossary of Osteopathic Terminology were reported [42]. Moreover, there is a common trend to use a “black box design” to consider osteopathic manipulative treatment as that set (not exactly reproducible) of diagnostic tests and techniques designed for patient treatment. This method is much more linked to everyday clinical reality, in which clinicians summarize approaches for administering person-centred osteopathic care [48].

Regarding osteopathic management in complex scenarios, it has been reported that person-centred osteopathic care is described as including tailored and symptom-based approaches [49]. Both whole body and segmental strategies are tailored to patients’ needs and individual responsiveness to the different types of osteopathic touch administered on regions of interest for both patients and osteopaths, i.e., SD. According to the structure/function models, the aim is to improve postural control, modulate autonomic neural overload, improve gastrointestinal function, breathing patterns, drainage and supply of body fluids, control stress components and augment reaction to biopsychosocial stressors [50,51]. Symptom-based approaches are considered in the treatment plan, i.e., administering appropriate techniques according to research studies conducted in a similar clinical context, not necessarily focusing osteopathic touch on SD. A possible explanation of the different percentages of focusing on SD in the available studies could be related to the aims of the interventions. In some cases, depending on person-centred tenets, the osteopathic approaches are more concentrated on improving and maintaining health and individual adaptability to the environment. In other studies, the strategy focuses on symptoms related to the patient’s chief complaint to deliver an evidence-informed practice.

In the present scoping review, we assumed that osteopathic touch is focused on SD of the musculoskeletal body framework interacting with self-regulation. The studies included in this review confirm that osteopathic practitioners not only work in the prevention field but also with a fragile population, such as in the pediatric field [11,12,52] or in patients with different pathologies [9,10,53,54].

Our results also confirm that the osteopathic patient demographics include a wide range of the general population, i.e., ranging from children to working-age adults and older adults, as reported in other studies [36]. Although studies in children have considered DS, professionals need to adapt the SD-based approach for infants and children. For example, one of the classical palpatory findings based on tenderness is not helpful in neonatal management because it is not possible to have feedback from newborns [55].

With respect to the time frame, the duration and frequency of osteopathic treatments were consistent with the data summarized in the osteopathic guidelines [8] and in a recent systematic review [56]. The differences in duration between 15 min to 1 h could be related to the different focus of the osteopathic intervention administered in the studies included in this review, i.e., a complex person-centred osteopathic intervention requires assessing regions of interest in the osteopathic patient dyadic relationship [24]. The shared decision-making process, which is based on verbal and non-verbal communication (i.e., on proximity and touch) usually takes a considerable amount of time [51]. Conversely, the administration of a single technique or a standardised approach is faster.

Differences in terms of treatment plan duration and follow-up could depend on the different outcomes of the studies (i.e., the experience of pain or functional status), primarily because the subjective experience of pain might respond to treatment sooner than function [42]. Moreover, the improvement of individual agency and comorbid psychological factors in patients with physical conditions and a high allostatic load requires a complex personalised intervention rather than a single technique [57].

Half of the selected studies reported the use of SD to guide OMT and most of them did not use standardised treatment protocols. Moreover, although the “dosage” of treatments proposed indicates the non-uniformity of osteopathic clinical procedures in research, there was no evidence that an appropriate “posology” was proposed for each patient’s condition. The subjectivity of person-centred care requires continuous adaptations in assessment and treatment to support human variability; however, the lack of standards can complicate research and, therefore, the understanding of the mechanisms of action in osteopathy. Furthermore, the heterogeneity of studies reveals a confusing synthesis of information that does not contribute to patient safety and the generalisation of findings leading to treatment efficacy. For this reason, the Template for Intervention Description and Replication (TIDieR) [58] was developed. This is a checklist which ensures that the characteristics of the intervention used are reported in the best possible way. Gerard Alvarez et al. [59] suggested that the TIDieR should be used in the field of manual and manipulative therapies, where the proposed therapies are administered with a high degree of customisation and variability. To overcome these difficulties, in 2007 the American Academy of Orthopaedic Manual Physical Therapists [60] set up a task force to standardise terminology in manual therapy, starting with the nomenclature of manipulative intervention, in order to make it more internationally usable. Chaitow [61] tried to classify the forces of the techniques used to characterise them with different descriptors (i.e., direction, force, velocity, amplitude, etc.).

To the best of our knowledge, this is the first literature analysis aimed to investigate the role and use of SD by considering many studies. The relevance of SD in osteopathic practice varies from country to country. Depending on the specific SD, the assessment and treatment modality should consider a whole body, loco-regional or segmental approach. SD should be considered as a clinical value that assists in the clinical assessment and guides the decision-making process of osteopathic practitioners.

According to recent reviews [6,9,53,56] a large amount of heterogeneity emerged from osteopathic research, particularly concerning treatment modalities. As previously discussed, authors of the included studies considered various protocols in terms of manipulative techniques, period of treatments, frequency and intensity of sessions and, lastly, adopted rationale toward SD. This fact represents an important barrier both to demonstrate the osteopathic manipulation effectiveness and to provide specific guidelines to clinicians. From another point of view, it should be considered how osteopathic medicine is traditionally characterized by a tailored approach on the single person rather than to the disease [1,2]; thus, a complete standardization becomes difficult, in the clinic as well as in research.

### Strengths and Limitations

We acknowledge some limitations. First, since this study was a scoping review, we evaluate the methods of the included studies about the use of osteopathic SD and not the effectiveness of the osteopathy, that should be investigated in further studies. A large study team screened the number of studies and other sources for potential inclusion in the scoping review. In fact, more than 20 h per person were invested to train the team. This review does not provide a synthesised result but instead provides an overview of the available literature on an important topic for the osteopathic profession. Like other studies, this review is at risk of bias from different sources. Another limitation is the potential gap between osteopathic clinical practice and the osteopathic methods reported in the literature; indeed, future studies should use qualitative methods to highlight osteopaths’ attitudes and preferences during clinical practice.

## 5. Conclusions

This review provides an overview of what is known about the concepts of SD in a worldwide context sharing methodological steps to develop osteopathic care theoretical models. The background provided above could involve many osteopathic researchers, educators, and practitioners in a consensus conference to work towards consistency, plausibility, generalisability, relevance and expected applicability of SD. Further studies should be designed to better understand why and how to choose the different assessment and intervention modalities to approach SD.

## Data Availability

The data presented in this study are available on request from the corresponding author.

## Supplementary Materials

The following are available online at https://www.mdpi.com/article/10.3390/healthcare10010028/s1, Table S1: Search Strategy; Table S2: References of the included studies in the review; Table S3: Preferred Reporting Items for Systematic reviews and Meta-Analyses extension for Scoping Reviews (PRISMA-ScR) Checklist.
