# Supplementary material for: International Overview of Somatic Dysfunction Assessment and Treatment in Osteopathic Research: A Scoping Review
Source: Healthcare (Basel). 2021 Dec 24;10(1):28. doi: 10.3390/healthcare10010028 (PMC8775713; doi:10.3390/healthcare10010028)
Supplement: Supplementary file 1 [file healthcare-10-00028-s001.zip › healthcare-1488769-supplementary.pdf]

Supplementary File

Table S1. Search Strategy

The 2 following strings have been inserted in the databases.	
"somatic dysfunction" OR "osteopathic dysfunction" OR "osteopathic lesion" OR "osteopathy" OR "osteopathic manipulation" OR "osteopathic manipulative treatment" OR "osteopathic treatment" OR "spinal manipulation" OR "visceral manipulation" OR "TART criteria" OR "osteopathic medicine" OR "myofascial release"	"craniosacral osteopathy" OR "craniosacral therapy" OR "craniosacral treatment" OR "craniosacral therapy" OR "craniosacral manipulation" OR "cranio-sacral osteopathy" OR "cranio-sacral therapy" OR "craniosacral treatment" OR "cranio-sacral therapy" OR "cranio-sacral manipulation" OR "cranial osteopathy", "cranial therapy" OR "cranial treatment" OR "cranial therapy" OR "cranial manipulation".

Table S2. References of the included studies in the review

1. Accorsi A, Lucci C, Di Mattia L, Granchelli C, Barlafante G, Fini F, et al. Effect of osteopathic manipulative therapy in the attentive performance of children with attention-deficit/hyperactivity disorder. J Am Osteopath Assoc. 2014;114(5):374–81.
2. Accorsi A, Lucci C, Pizzolorusso G, Tubaldi L, Cerritelli F, Perri FP. Neonatology-osteopathy (NE-O) study: RCT on the effect of osteopathic manipulative treatment on LOS. Arch Dis Child. 2012;97((Accorsi A.; Pizzolorusso G.; Cerritelli F.) European Institute for Evidence Based Osteopathic Medicine, Pescara, Italy):A277–8.
3. Acquati A, Uberti S, Aquino A, Cerasetti E, Castagna C, Rovere-Querini P, et al. Do empathic osteopaths achieve better clinical results? An observational feasibility study. International Journal of Osteopathic Medicine. 1 giugno 2019;32:2–6.
4. Adragna V, Piazzolla S, Lo Voi G. Osteopathic manipulative treatment of headache in a polytrauma patient: Case report. Neurol Sci. 2015;36(1):S175–6.
5. Albers J, Jäkel A, Wellmann K, von Hehn U, Schmidt T. Effectiveness of 2 Osteopathic Treatment Approaches on Pain, Pressure-Pain Threshold, and Disease Severity in Patients with Fibromyalgia: A Randomized Controlled Trial. Complement Med Res. 2018;25(2):122–8.
6. Alexandre K, Channell MK. Osteopathic approach to the treatment of a patient with an atypical presentation of coccydynia. J Am Osteopath Assoc. 2019;119(6):395–400.
7. Altınbilek T, Murat S, Yumuşakhuylu Y, İçağasıoğlu A. Osteopathic manipulative treatment improves function and relieves pain in knee osteoarthritis: A single-blind, randomized-controlled trial. Turk J Phys Med Rehabil. giugno 2018;64(2):114–20.
8. Anderson RE, Seniscal C. A comparison of selected osteopathic treatment and relaxation for tension-type headaches. Headache. settembre 2006;46(8):1273–80.
9. Andersson GB, Lucente T, Davis AM, Kappler RE, Lipton JA, Leurgans S. A comparison of osteopathic spinal manipulation with standard care for patients with low back pain. N Engl J Med. 4 novembre 1999;341(19):1426–31.
10. Andreoli E, Troiani A, Tucci V, Barlafante G, Cerritelli F, Pizzolorusso G, et al. Osteopathic manipulative treatment of congenital talipes equinovarus: a case report. J Bodyw Mov Ther. gennaio 2014;18(1):4–10.
11. Apoznanski TE, Flaum TB. Role of osteopathic manipulative treatment in a dynamic case of Parkinson disease and levodopa-induced dyskinesia: A case report. AAO J. 2015;25(1):21–4.
12. Aquino A, Perini M, Cosmai S, Zanon S, Pisa V, Castagna C, et al. Osteopathic Manipulative Treatment Limits Chronic Constipation in a Child with Pitt-Hopkins Syndrome. Case Rep Pediatr [Internet]. 2017 [citato 1 aprile 2021];2017. Disponibile su: https://www.ncbi.nlm.nih.gov/pmc/articles/PMC5306969/
13. Arab AM, Nourbakhsh MR. The effect of cranial osteopathic manual therapy on somatic tinnitus in individuals without otic pathology: Two case reports with one year follow up. Int J Ostheopath Med. 2014;17(2):123–8.
14. Arienti C, Daccò S, Piccolo I, Redaelli T. Osteopathic manipulative treatment is effective on pain control associated to spinal cord injury. Spinal Cord. aprile 2011;49(4):515–9.
15. Arienti C, Bosisio T, Ratti S, Miglioli R, Negrini S. Osteopathic Manipulative Treatment Effect on Pain Relief and Quality of Life in Oncology Geriatric Patients: A Nonrandomized Controlled Clinical Trial. Integr Cancer Ther. dicembre 2018;17(4):1163–71.
16. Atay F, Bayramlar K, Sarac ET. Effects of Craniosacral Osteopathy in Patients with Peripheral Vestibular Pathology. ORL J Otorhinolaryngol Relat Spec. 2021;83(1):7–13.
17. Attali T, Bouchoucha M, Benamouzig R. Treatment of refractory irritable bowel syndrome with visceral osteopathy: short-term and long-term results of a randomized trial. Journal of digestive diseases. 2013;14(12):654-661.
18. Auger K, Shedlock G, Coutinho K, Myers NE, Lorenzo S. Effects of osteopathic manipulative treatment and bio-electromagnetic energy regulation therapy on lower back pain. J Osteopath Med. 2 marzo 2021;

19. Baisakhiya N. Efficacy of osteopathic manipulative treatment in the patient of chronic rhinosinusitis: a case report. Clinical rhinology. 2018;112-3:58-60.

20. Baker JP, Baker CD. Osteopathic manipulative treatment for Lyme disease-induced Bell's palsy: A case study. AAO J. 2013;23(1):12–5.

21. Baltazar GA, Kolwitz C, Petrone P, Stright A, Joseph D. Osteopathic Manipulative Treatment Relieves Post-concussion Symptoms in a Case of Polytrauma. Cureus [Internet]. 2020 [citato 1 aprile 2021];12(3). Disponibile su: https://www.ncbi.nlm.nih.gov/pmc/articles/PMC7164692/

22. Baltazar GA, Betler MP, Akella K, Khatri R, Asaro R, Chendrasekhar A. Effect of osteopathic manipulative treatment on incidence of postoperative ileus and hospital length of stay in general surgical patients. J Am Osteopath Assoc. marzo 2013;113(3):204–9.

23. Barni A, Zecchillo D, Uberti S, Ratti S. Osteopathic manipulative treatment in a paediatric patient with oesophageal atresia and tracheo-oesophageal fistula. Case Rep Gastroenterol. 2019;13(1):178–84.

24. Batt J, Neeki MM. Osteopathic manipulative treatment in tarsal somatic dysfunction: a case study. J Am Osteopath Assoc. novembre 2013;113(11):857–61.

25. Belsky JA, Wolf K, Setty BA. A Case of Resolved Vincristine-Induced Constipation Following Osteopathic Medicine in a Patient With Infantile Fibrosarcoma. J Am Osteopath Assoc. 1 ottobre 2020;120(10):691–5.

26. Belvaux A, Bouchoucha M, Benamouzig R. Osteopathic management of chronic constipation in women patients. Results of a pilot study. Clin Res Hepatol Gastroenterol. ottobre 2017;41(5):602–11.

27. Bendixen K, Beinlich A, Beck B, Hashmi N IV, Craig A. Pilot study assessing the effect of osteopathic manipulative treatment (Omt) on length of stay in neonates after therapeutic hypothermia. J Am Osteopath Assoc. 2021;121(1):97–104.

28. Benjamin JG, Moran RW, Plews DJ, Kilding AE, Barnett LE, Verhoeff WJ, et al. The effect of osteopathic manual therapy with breathing retraining on cardiac autonomic measures and breathing symptoms scores: A randomised wait-list controlled trial. Journal of Bodywork and Movement Therapies. 1 luglio 2020;24(3):282–92.

29. Bennett S, Macfarlane C, Vaughan B. The Use of Osteopathic Manual Therapy and Rehabilitation for Subacromial Impingement Syndrome: A Case Report. Explore (NY). ottobre 2017;13(5):339–43.

30. Berkowitz MR. Application of osteopathic manipulative treatment to a patient with unremitting chest pain and shortness of breath undergoing "Rule-Out Myocardial Infarction" protocol for one week. International Journal of Osteopathic Medicine. 1 giugno 2012;15(2):73–7.

31. Berkowitz MR. Application of osteopathy in the cranial field to treat left superior homonymous hemianopsia. International Journal of Osteopathic Medicine. 1 giugno 2014;17(2):119–22.

1. 32.

2. Bockenhauer S, Julliard K, Lo K, Huang E, Sheth A. Quantifiable effects of osteopathic manipulative techniques on patients with chronic asthma. Journal of the American Osteopathic Association. 2002;102(7):371-5; discussion 375.

32. Bordoni B, Marelli F, Morabito B, Sacconi B. Osteopathic treatment in a patient with left-ventricular assist device with left brachialgia: a case report. Int Med Case Rep J. 13 gennaio 2017;10:19–23.

33. Bordoni B, Morabito B, Simonelli M, Nicoletti L, Rinaldi R, Tobbi F, et al. Osteopathic approach with a patient undergoing cardiac transplantation: the five diaphragms. Int Med Case Rep J. 2019;12:303–8.

34. Brolinson PG, Smolka M, Rogers M, Sukpraprut S, Goforth MW, Tilley G, et al. Precompetition manipulative treatment and performance among Virginia Tech athletes during 2 consecutive football seasons: a preliminary, retrospective report. J Am Osteopath Assoc. settembre 2012;112(9):607–15.

35. Brugman R, Fitzgerald K, Fryer G. The effect of Osteopathic Treatment on Chronic Constipation – A Pilot Study. International Journal of Osteopathic Medicine. 1 marzo 2010;13(1):17–23.

36. Brumm LF, Janiski C, Balawender JL, Feinstein A. Preventive osteopathic manipulative treatment and stress fracture incidence among collegiate cross-country athletes. J Am Osteopath Assoc. dicembre 2013;113(12):882–90.

37. Burnham T, Higgins D, Burnham R, Heath D. Effectiveness of osteopathic manipulative treatment for carpal tunnel syndrome: a pilot project. Journal of the american osteopathic association. 2015;115(3):138-148.

38. Buscemi A, Pennisi V, Rapisarda A, Pennisi A, Coco M. Efficacy of osteopathic treatment in patients with stable moderate-to-severe chronic obstructive pulmonary disease: a randomized controlled pilot study. Journal of complementary & integrative medicine [Internet]. 2019;17(1). Disponibile su: https://www.cochranelibrary.com/central/doi/10.1002/central/CN-01989969/full

39. Buscemi A, Cannatella M, Lutrario P, Rapisarda A, Di Gregorio G, Coco M. Effects of osteopathic treatment on postural equilibrium evaluated through a stabilometric platform: A randomized and controlled study. J Funct Morphol Kinesiol [Internet]. 2017;2(2). Disponibile su: https://www.scopus.com/inward/record.uri?eid=2-s2.0-85059736598&doi=10.3390%2fjfmk2020018&partnerID=40&md5=5cf1c67c950b93e51210a1f0410c55b7

40. Buscemi A, Petralia MC, Ramaci T, Rapisarda A, Provazza C, Di Corrado D, et al. Ergojump evaluation of the explosive strength in volleyball athletes pre- and post-fascial treatment. Exp Ther Med. agosto 2019;18(2):1470–6.

41. Carnes D, Bright P, Brownhill K, Carroll K, Engel R, Grace S, et al. Crying Unsettled and disTressed Infants Effectiveness Study of osteopathic care (CUTIES trial): Pragmatic randomised superiority trial protocol. International Journal of Osteopathic Medicine. 1 dicembre 2020;38:31–8.

42. Carnevali L, Cerritelli F, Guolo F, Sgoifo A. Osteopathic Manipulative Treatment and Cardiovascular Autonomic Parameters in Rugby Players: A Randomized, Sham-Controlled Trial. J Manipulative Physiol Ther. 9 gennaio 2021;

43. Castillo I, Wolf K, Rakowsky A. Concussions and Osteopathic Manipulative Treatment: An Adolescent Case Presentation. J Am Osteopath Assoc. marzo 2016;116(3):178–81.

44. Castro-Sanchez. Benefits of Craniosacral Therapy in Patients with Chronic Low Back Pain: a Randomized Controlled Trial. Journal of alternative and complementary medicine 22 (8) (pp 650-657), 2016 Date of publication: august 2016 [Internet]. 2016; Disponibile su: https://www.cochranelibrary.com/central/doi/10.1002/central/CN-01193989/full

45. Cerritelli F, Pizzolorusso G, Renzetti C, Cozzolino V, D'Orazio M, Lupacchini M, et al. A multicenter, randomized, controlled trial of osteopathic manipulative treatment on preterms. PloS one. 2015;10(5):e0127370.

46. Cerritelli F, Ginevri L, Messi G, Renzetti C, Cozzolino V, Barlafante G, et al. Effectiveness of osteopathic manipulative treatment in migraine: Three-armed randomized controlled trial. J Neurol. 2013;260((Cerritelli F.; Ginevri L.; Messi G.; Renzetti C.; Cozzolino V.; Barlafante G.; Foschi N.) European Institute for Evidence Based Osteopathic Medicine (Pescara, IT); Accademia Italiana Osteopatia Tradizionale (Pescara, IT); Ancona's Hospital (Ancona, IT)):S159–60.

47. Cerritelli F, Cardone D, Pirino A, Merla A, Scoppa F. Does Osteopathic Manipulative Treatment Induce Autonomic Changes in Healthy Participants? A Thermal Imaging Study. Front Neurosci. 2020;14:887.

48. Cerritelli F, Carinci F, Pizzolorusso G, Turi P, Renzetti C, Pizzolorusso F, et al. Osteopathic manipulation as a complementary treatment for the prevention of cardiac complications: 12-Months follow-up of intima media and blood pressure on a cohort affected by hypertension. Journal of Bodywork and Movement Therapies. 1 gennaio 2011;15(1):68–74.

49. Cerritelli F, Chiacchiaretta P, Gambi F, Perrucci MG, Barassi G, Visciano C, et al. Effect of manual approaches with osteopathic modality on brain correlates of interoception: an fMRI study. Sci Rep. 21 febbraio 2020;10(1):3214.

50. Cerritelli F, Pizzolorusso G, Ciardelli F, La Mola E, Cozzolino V, Renzetti C, et al. Effect of osteopathic manipulative treatment on length of stay in a population of preterm infants: a randomized controlled trial. BMC Pediatr. 26 aprile 2013;13:65.

51. Chin J, Qiu W, Lomiguen CM, Volokitin M. Osteopathic Manipulative Treatment in Tension Headaches. Cureus [Internet]. [citato 1 aprile 2021];12(12). Disponibile su: https://www.ncbi.nlm.nih.gov/pmc/articles/PMC7799179/

52. Chmielewski R, Pena N, Capalbo G. Osteopathic manipulative treatment of pes anserine bursitis using the triple technique: A case report. AAO J. 2013;23(1):34–8.

53. Chown M, Whittamore L, Rush M, Allan S, Stott D, Archer M. A prospective study of patients with chronic back pain randomised to group exercise, physiotherapy or osteopathy. Physiotherapy. 2008;94(1):21-28.

54. Chvetzoff G, Berthier A, Blanc E, Bourne Branchu V, Millaret A, Cropet C, et al. Osteopathy for chronic pain after breast cancer surgery: A monocentric randomised study. Bull Cancer. 2019;106(5):436–46.

55. Cicchitti L, Di Lelio A, Barlafante G, Cozzolino V, Di Valerio S, Fusilli P, et al. Osteopathic Manipulative Treatment in Neonatal Intensive Care Units. Med Sci (Basel) [Internet]. 24 giugno 2020 [citato 1 aprile 2021];8(2). Disponibile su: https://www.ncbi.nlm.nih.gov/pmc/articles/PMC7353589/

56. Clark BC, Walkowski S, Conatser RR, Eland DC, Howell JN. Muscle functional magnetic resonance imaging and acute low back pain: a pilot study to characterize lumbar muscle activity asymmetries and examine the effects of osteopathic manipulative treatment. Osteopath Med Prim Care. 27 agosto 2009;3:7.

57. Cohen-Lewe A. Osteopathic manipulative treatment for colonic inertia. J Am Osteopath Assoc. marzo 2013;113(3):216–20.

58. Cordano C, Armezzani A, Veroni J, Pardini M, Sassos D, Infante MT, et al. Osteopathic manipulative therapy and multiple sclerosis: A proof-of-concept study. J Am Osteopath Assoc. 2018;118(8):531–6.

59. Cromeens B, Gamber R. Osteopathic manipulative treatment in a patient with idiopathic dysautonomia: a case presentation. Osteopathic Family Physician. 1 settembre 2010;2(5):144–7.

60. Crow WT. Manipulative treatment for idiopathic impotence in a 24-year-old water polo player. International Journal of Osteopathic Medicine. 1 giugno 2006;9(2):66–71.

61. Cruser des A, Maurer D, Hensel K, Brown SK, White K, Stoll ST. A randomized, controlled trial of osteopathic manipulative treatment for acute low back pain in active duty military personnel. J Man Manip Ther. febbraio 2012;20(1):5–15.

62. Cuccia AM, Caradonna C, Annunziata V, Caradonna D. Osteopathic manual therapy versus conventional conservative therapy in the treatment of temporomandibular disorders: a randomized controlled trial. J Bodyw Mov Ther. aprile 2010;14(2):179–84.

63. D'Ippolito M, Tramontano M, Buzzi MG. Effects of osteopathic manipulative therapy on pain and mood disorders in patients with high-frequency migraine. J Am Osteopath Assoc. 2017;117(6):365–9.

64. Dade MM, Broecker JD. Myofascial Release for Vulvar Pain and Pubic Shear After a Straddle Injury in a 3-Year-Old Girl. J Am Osteopath Assoc. 1 novembre 2019;119(11):768–71.

65. Daraï C, Deboute O, Zacharopoulou C, Laas E, Canlorbe G, Belghiti J, et al. Impact of osteopathic manipulative therapy on quality of life of patients with deep infiltrating endometriosis with colorectal involvement: results of a pilot study. Eur J Obstet Gynecol Reprod Biol. maggio 2015;188:70–3.

66. de Oliveira Meirelles F, de Oliveira Muniz Cunha JC, da Silva EB. Osteopathic manipulation treatment versus therapeutic exercises in patients with chronic nonspecific low back pain: A randomized, controlled and double-blind study. J Back Musculoskelet Rehabil. 2020;33(3):367–77.

67. Degenhardt BF, Darmani NA, Johnson JC, Towns LC, Rhodes DCJ, Trinh C, et al. Role of osteopathic manipulative treatment in altering pain biomarkers: a pilot study. J Am Osteopath Assoc. settembre 2007;107(9):387–400.

68. Degenhardt BF, Kuchera ML. Osteopathic evaluation and manipulative treatment in reducing the morbidity of otitis media: a pilot study. J Am Osteopath Assoc. giugno 2006;106(6):327–34.

69. Deodato M, Guolo F, Monticco A, Fornari M, Manganotti P, Granato A. Osteopathic Manipulative Therapy in Patients With Chronic Tension-Type Headache: a Pilot Study. Journal of the American Osteopathic Association [Internet]. 2019; Disponibile su: https://www.cochranelibrary.com/central/doi/10.1002/central/CN-01980630/full

70. DiFrancisco-Donoghue J, Apoznanski T, de Vries K, Jung M-K, Mancini J, Yao S. Osteopathic manipulation as a complementary approach to Parkinson's disease: A controlled pilot study. NeuroRehabilitation. 2017;40(1):145–51.

71. Dixon L, Fotinos K, Sherifi E, Lokuge S, Fine A, Furtado M, et al. Effect of osteopathic manipulative therapy on generalized anxiety disorder. J Am Osteopath Assoc. 2020;120(3):133–43.

72. Dugailly P-M, Fassin S, Maroye L, Evers L, Klein P, Feipel V. Effect of a general osteopathic treatment on body satisfaction, global self perception and anxiety: a randomized trial in asymptomatic female students. International journal of osteopathic medicine. 2014;17(2):94-101.

73. Duncan B, McDonough-Means S, Worden K, Schnyer R, Andrews J, Meaney FJ. Effectiveness of osteopathy in the cranial field and myofascial release versus acupuncture as complementary treatment for children with spastic cerebral palsy: A pilot study. J Am Osteopath Assoc. 2008;108(10):559–70.

74. Edwards DJ, Toutt C. An evaluation of osteopathic treatment on psychological outcomes with patients suffering from chronic pain: A prospective observational cohort study collected through a health and well-being academy. Health Psychol Open [Internet]. 10 maggio 2018 [citato 1 aprile 2021];5(1). Disponibile su: https://www.ncbi.nlm.nih.gov/pmc/articles/PMC5952292/

75. Eisenhart AW, Gaeta TJ, Yens DP. Osteopathic manipulative treatment in the emergency department for patients with acute ankle injuries. J Am Osteopath Assoc. settembre 2003;103(9):417–21.

76. Espí-López G-V, Ruescas-Nicolau M-A, Nova-Redondo C, Benítez-Martínez JC, Dugailly P-M, Falla D. Effect of Soft Tissue Techniques on Headache Impact, Disability, and Quality of Life in Migraine Sufferers: A Pilot Study. J Altern Complement Med. 2018;24(11):1099–107.

77. Fernandes WVB, Blanco CR, Politti F, de Cordoba Lanza F, Lucareli PRG, Corrêa JCF. The effect of a six-week osteopathic visceral manipulation in patients with non-specific chronic low back pain and functional constipation: study protocol for a randomized controlled trial. Trials [Internet]. 2 marzo 2018 [citato 1 aprile 2021];19. Disponibile su: https://www.ncbi.nlm.nih.gov/pmc/articles/PMC5833057/

78. Fleming RK, Snider KT, Blanke KJ, Johnson JC. The effect of osteopathic manipulative treatment on length of stay in posterolateral postthoracotomy patients: A retrospective case note study. International Journal of Osteopathic Medicine. 1 giugno 2015;18(2):88–96.

79. Florance B, Frin G, Dainese R, Nébot-Vivinus M, Marine Barjoan E, Marjoux S, et al. Osteopathy improves the severity of irritable bowel syndrome: a pilot randomized sham-controlled study. European journal of gastroenterology & hepatology. 2012;24(8):944-949.

80. Fraix M. Osteopathic treatment for vertigo: A pilot study. Osteopath Med. 2011;12(1):4–11.

81. Fraix M. Osteopathic manipulative treatment and vertigo: a pilot study. PM R. luglio 2010;2(7):612–8.

82. Frymann VM, Carney RE, Springall P. Effect of osteopathic medical management on neurologic development in children. J Am Osteopath Assoc. giugno 1992;92(6):729–44.

83. Fuller DB. Osteopathic Approach to the Treatment of a Patient With Idiopathic Iliohypogastric Neuralgia. J Am Osteopath Assoc. 1 dicembre 2020;120(12):907–12.

84. Galindez-Ibarbengoetxea X, Setuain I, Ramírez-Velez R, Andersen L, González-Izal M, Jauregi A, et al. Immediate Effects of Osteopathic Treatment Versus Therapeutic Exercise on Patients With Chronic Cervical Pain. Alternative therapies in health and medicine. 2018;24(3):24-32.

85. Gamber RG; Shores JH; Russo DP; Jimenez C; Rubin BR. Osteopathic manipulative treatment in conjunction with medication relieves pain associated with fibromyalgia syndrome: results of a randomized clinical pilot project. The Journal of the American Osteopathic Association 2002 Jun;102(6):321-325. 2002;

86. Gelfman DM. Osteopathic Manipulation in Treatment of Musculoskeletal Chest Pain. Am J Med. giugno 2017;130(6):618.

87. Genese JS. Osteopathic manipulative treatment for facial numbness and pain after whiplash injury. J Am Osteopath Assoc. luglio 2013;113(7):564–7.

88. Gesslbauer C, Vavti N, Keilani M, Mickel M, Crevenna R. Effectiveness of osteopathic manipulative treatment versus osteopathy in the cranial field in temporomandibular disorders - a pilot study. Disability and rehabilitation. 2018;40(6):631-636.

89. Gibson T, Grahame R, Harkness J, Woo P, Blagrave P, Hills R. Controlled comparison of short-wave diathermy treatment with osteopathic treatment in non-specific low back pain. Lancet. 1 giugno 1985;1(8440):1258–61.

90. Gilliss AC, Swanson RL, Janora D, Venkataraman V. Use of osteopathic manipulative treatment to manage compensated trendelenburg gait caused by sacroiliac somatic dysfunction. J Am Osteopath Assoc. 2010;110(2):81–6.

91. Goodkin MB, Bellew LJ. Osteopathic manipulative treatment for postural orthostatic tachycardia syndrome. J Am Osteopath Assoc. novembre 2014;114(11):874–7.

92. Goyal K, Goyal M, Narkeesh K, John Samuel A, Sharma S, Chatterjee S, et al. The effectiveness of osteopathic manipulative treatment in an abnormal uterine bleeding related pain and health related quality of life (HR-QoL) – A case report. Journal of Bodywork and Movement Therapies. 1 luglio 2017;21(3):569–73.

93. Goyal M, Goyal K, Narkeesh K, Samuel AJ, Arumugam N, Chatterjee S, et al. Efficacy of osteopathic manipulative treatment approach in the patient with pulmonary fibrosis in critical care outpatient department. Indian J Crit Care Med. 2017;21(7):469–72.

94. Goyal M, Goyal K, Narkeesh K, Samuel AJ, Arumugam N. Osteopathic manipulative treatment for post mastectomy lymphedema: A case report. Int J Ostheopath Med. 2017;26:49–52.

95. Goyal M, Aggarwal A, Goyal K, Garg P. Effectiveness of Osteopathic Therapy in the Treatment of Oral Submucous Fibrosis. Contemp Clin Dent. 2017;8(1):145–7.

96. Gray RE, Kasper K. Osteopathic Manipulative Treatment as a Novel Way to Manage Postvasectomy Pain Syndrome. J Am Osteopath Assoc. 29 ottobre 2018;

97. Groisman S; Malysz T; de Souza da Silva L; Rocha Ribeiro Sanches T; Camargo Bragante K; Locatelli F; Pontel Vigolo C; Vaccari S; Homercher Rosa Francisco C; Monteiro Steigleder S; Jotz GP. Osteopathic manipulative treatment combined with exercise improves pain and disability in individuals with non-specific chronic neck pain: a pragmatic randomized controlled trial [with consumer summary]. Journal of Bodywork and Movement Therapies 2020 Apr;24(2):189-195. 2020;

98. Guernsey DT 3rd, Leder A, Yao S. Resolution of Concussion Symptoms After Osteopathic Manipulative Treatment: A Case Report. J Am Osteopath Assoc. marzo 2016;116(3):e13-17.

99. Gugel MR, Johnston WL. Osteopathic manipulative treatment of a 27-year-old man after anterior cruciate ligament reconstruction. J Am Osteopath Assoc. giugno 2006;106(6):346–9.

100. Guiney PA, Chou R, Vianna A, Lovenheim J. Effects of osteopathic manipulative treatment on pediatric patients with asthma: a randomized controlled trial. J Am Osteopath Assoc. gennaio 2005;105(1):7–12.

101. Haiden N, Pimpel B, Kreissl A, Jilma B, Berger A. Does visceral osteopathic treatment accelerate meconium passage in very low birth weight infants?- A prospective randomized controlled trial. PloS one. 2015;10(4):e0123530.

102. Halimi M, Leder A, Mancini JD. Integration of Osteopathic Manual Treatments in Management of Cervical Dystonia with Tremor: A Case Series. Tremor Other Hyperkinet Mov (N Y). 2017;7:435.

103. Hasler C, Schmid C, Enggist A, Neuhaus C, Erb T. No effect of osteopathic treatment on trunk morphology and spine flexibility in young women with adolescent idiopathic scoliosis. J Child Orthop. giugno 2010;4(3):219–26.

104. Hastings V, McCallister AM, Curtis SA, Valant RJ, Yao S. Efficacy of Osteopathic Manipulative Treatment for Management of Postpartum Pain. J Am Osteopath Assoc. 1 agosto 2016;116(8):502–9.

105. Hayden C, Mullinger B. A preliminary assessment of the impact of cranial osteopathy for the relief of infantile colic. Complementary therapies in clinical practice. 2006;12(2):83-90.

106. Heineman K. Osteopathic manipulative treatment in the management of pediatric headache and orthodontic intervention: A case report. AAO J. 2018;28(1):15–8.

107. Heineman K. Osteopathic manipulative treatment in the management of biliary dyskinesia. J Am Osteopath Assoc. febbraio 2014;114(2):129–33.

108. Hensel KL, Buchanan S, Brown SK, Rodriguez M, Cruser des A. Pregnancy Research on Osteopathic Manipulation Optimizing Treatment Effects: the PROMOTE study. Am J Obstet Gynecol. gennaio 2015;212(1):108.e1-9.

109. Hensel KL, Pacchia CF, Smith ML. ACUTE IMPROVEMENT IN HEMODYNAMIC CONTROL AFTER OSTEOPATHIC MANIPULATIVE TREATMENT IN THE THIRD TRIMESTER OF PREGNANCY. Complement Ther Med. dicembre 2013;21(6):618–26.

110. Hensel K, Pacchia C, Smith M. Acute improvement in hemodynamic control after osteopathic manipulative treatment in the third trimester of pregnancy. Complementary therapies in medicine. 2013;21(6):618-626.

111. Herzaft-LeRoy J, Xhignesse M, Gaboury I. Assessment of the Efficacy of An Osteopathic Treatment in Infants with Biomechanical Impairments to Suckling. JoVE. 5 febbraio 2019;(144):58740.

112. Herzhaft-Le Roy J, Xhignesse M, Gaboury I. Efficacy of an Osteopathic Treatment Coupled With Lactation Consultations for Infants' Biomechanical Sucking Difficulties. J Hum Lact. febbraio 2017;33(1):165–72.

113. Hess NJ. Osteopathic manipulative treatment for nausea and vomiting following fine needle aspiration of the neck. AAO J. 2017;27(1):12–5.

114. Huard Y. Osteopathic treatment of chronic gonalgia in the elderly: Changes in pain and posture. AAO J. 2012;22(4):52–3.

115. Hubert D, Soubeiran L, Gourmelon F, Grenet D, Serreau R, Perrodeau E, et al. Impact of osteopathic treatment on pain in adult patients with cystic fibrosis--a pilot randomized controlled study. PLoS One. 2014;9(7):e102465.

116. Hundscheid HWC, Pepels MJAE, Engels LGJB, Loffeld RJLF. Treatment of irritable bowel syndrome with osteopathy: results of a randomized controlled pilot study. J Gastroenterol Hepatol. settembre 2007;22(9):1394–8.

117. Ignatowicz A, Berkowitz MR. Imaging evidence demonstrating effectiveness of osteopathic visceral manipulation techniques in treating pseudo-obstruction. AAO J. 2017;27(1):7–10.

118. Jackson C, Loveless B. The Use of Osteopathic Manipulative Medicine in the Management of Recurrent Mastitis. J Am Osteopath Assoc. 2020;120(12):921–5.

119. Jacq O, Arnulf I, Similowski T, Attali V. Upper airway stabilization by osteopathic manipulation of the sphenopalatine ganglion versus sham manipulation in OSAS patients: a proof-of-concept, randomized, crossover, double-blind, controlled study. BMC Complement Altern Med. 20 dicembre 2017;17(1):546.

120. Jardine W, Gillis C, Rutherford D. The effect of osteopathic manual therapy on the vascular supply to the lower extremity in individuals with knee osteoarthritis: a randomized trial. International journal of osteopathic medicine. 2012;15(4):125-133.

121. Jones AL, Lockwood MD. Osteopathic manipulative treatment in pregnancy and augmentation of labor: A case report. AAO J. 2008;18(1):27–9.

122. Joshua. Resolution of New Daily Persistent Headache After Osteopathic Manipulative Treatment. J Am Osteopath Assoc. marzo 2016;116(3):182–5.

123. Kant R, Berkowitz MR. Osteopathic manipulative treatment of pelvic dysfunction in a postpartum patient with co-morbid headaches: A case report. AAO J. 2014;24(1):8–11.

124. Kennard EJ, Lieberman J, Saaid A, Rolfe KJ. A Preliminary Comparison of Laryngeal Manipulation and Postural Treatment on Voice Quality in a Prospective Randomized Crossover Study. J Voice. novembre 2015;29(6):751–4.

125. Kilgore T, Malia M, Di Giacinto B, Minter S, Samies J. Adjuvant Lymphatic Osteopathic Manipulative Treatment in Patients With Lower-Extremity Ulcers: Effects on Wound Healing and Edema. J Am Osteopath Assoc. 1 dicembre 2018;118(12):798–805.

126. Kim BJ; Ahn J; Cho H; Kim D; Kim T; Yoon B. Rehabilitation with osteopathic manipulative treatment after lumbar disc surgery: a randomised, controlled pilot study [with consumer summary]. International Journal of Osteopathic Medicine 2015 Mar;18(3):181-188. 2015;

127. King AA, Cox J, Bhatia S, Snider KT. Characteristics and treatment of geriatric patients in an osteopathic neuromusculoskeletal medicine (ONMM) clinic. J Osteopath Med. 18 febbraio 2021;

128. Kirk L, Underwood M, Chappell L, Martins-Mendez M, Thomas P. The effect of osteopathy in the treatment of chronic low back pain – a feasibility study. International Journal of Osteopathic Medicine. 1 marzo 2005;8(1):5–11.

129. Korotkov K, Shelkov O, Shevtsov A, Mohov D, Paoletti S, Mirosnichenko D, et al. Stress reduction with osteopathy assessed with GDV electrophotonic imaging: effects of osteopathy treatment. J Altern Complement Med. marzo 2012;18(3):251–7.

130. Kramp ME. Combined manual therapy techniques for the treatment of women with infertility: Acase series. J Am Osteopath Assoc. 2012;112(10):680–4.

131. Lagrange A, Decoux D, Briot N, Hennequin A, Coudert B, Desmoulins I, et al. Visceral osteopathic manipulative treatment reduces patient reported digestive toxicities induced by adjuvant chemotherapy in breast cancer: A randomized controlled clinical study. European Journal of Obstetrics & Gynecology and Reproductive Biology. 1 ottobre 2019;241:49–55.

132. Lancaster DG, Crow WT. Osteopathic manipulative treatment of a 26-year-old woman with Bell's palsy. J Am Osteopath Assoc. maggio 2006;106(5):285–9.

133. Lessard S, Gagnon I, Trottier N. Exploring the impact of osteopathic treatment on cranial asymmetries associated with nonsynostotic plagiocephaly in infants. Complementary therapies in clinical practice. 2011;17(4):193-198.

134. Lewis DD. Osteopathic manipulative treatment for chronic neck pain, headaches, and referred pain in a patient with myofascial pain syndrome: A case report. AAO J. 2017;27(2):11–4.

135. Licciardone JC, Kearns CM. Somatic dysfunction and its association with chronic low back pain, back-specific functioning, and general health: Results from the osteopathic trial. J Am Osteopath Assoc. 2012;112(7):420–8.

136. Licciardone JC, Kearns CM, Hodge LM, Bergamini MV. Associations of cytokine concentrations with key osteopathic lesions and clinical outcomes in patients with nonspecific chronic low back pain: results from the OSTEOPATHIC Trial. J Am Osteopath Assoc. 2012;112(9):596–605.

137. Licciardone JC, Minotti DE, Gatchel RJ, Kearns CM, Singh KP. Osteopathic manual treatment and ultrasound therapy for chronic low back pain: A randomized controlled trial. Ann Fam Med. 2013;11(2):122–9.

138. Licciardone JC, Stoll ST, Fulda KG, Russo DP, Siu J, Winn W, et al. Osteopathic manipulative treatment for chronic low back pain: A randomized controlled trial. Spine. 2003;28(13):1355–62.

139. Licciardone J, Buchanan S, Hensel K, King H, Fulda K, Stoll S. Osteopathic manipulative treatment of back pain and related symptoms during pregnancy: a randomized controlled trial. American journal of obstetrics and gynecology. 2010;202(1):43.e1-8.

140. Licciardone J, Stoll S, Cardarelli K, Gamber R, Swift J, Winn W. A randomized controlled trial of osteopathic manipulative treatment following knee or hip arthroplasty. Journal of the American Osteopathic Association. 2004;104(5):193-202.

141. Licciardone J, Stoll S, Fulda K, Russo D, Siu J, Winn W, et al. Osteopathic manipulative treatment for chronic low back pain: a randomized controlled trial. Spine. 2003;28(13):1355-1362.

142. Licciardone JC, Nelson KE, Glonek T, Sleszynski SL, Cruser des A. Osteopathic manipulative treatment of somatic dysfunction among patients in the family practice clinic setting: a retrospective analysis. J Am Osteopath Assoc. dicembre 2005;105(12):537–44.

143. Litman RG. Osteopathic manipulative treatment of somatic dysfunction as an integral component in the care of patients with chronic medical disease: A thirty-month study in rural Appalachia. AAO J. 2012;22(3):26–51.

144. Lombardini R, Marchesi S, Collebrusco L, Vaudo G, Pasqualini L, Ciuffetti G, et al. The use of osteopathic manipulative treatment as adjuvant therapy in patients with peripheral arterial disease. Man Ther. 2009;14(4):439–43.

145. Lorenzo S, Nicotra CM, Mentreddy AR, Padia HJ, Stewart DO, Hussein MO, et al. Assessment of Pulmonary Function After Osteopathic Manipulative Treatment vs Standard Pulmonary Rehabilitation in a Healthy Population. J Am Osteopath Assoc. 11 febbraio 2019;

146. Lund GC, Edwards G, Medlin B, Keller D, Beck B, Carreiro JE. Osteopathic manipulative treatment for the treatment of hospitalized premature infants with nipple feeding dysfunction. J Am Osteopath Assoc. 2011;111(1):44–8.

147. Maggiani A; Tremolizzo L; Valentina AD; Mapelli L; Sosio S; Milano V; Bianchi M; Badi F; Lavazza C; Grandini M; Corna G; Prometti P; Lunetta C; Riva N; Ferri A; Lanfranconi F; for the ME&SLA Study. Osteopathic manual treatment for amyotrophic lateral sclerosis: a feasibility pilot study. The Open Neurology Journal 2016 Aug 26;10:59-66. 2016;

148. Manzotti A, Cerritelli F, Lombardi E, La Rocca S, Chiera M, Galli M, et al. Effects of osteopathic treatment versus static touch on heart rate and oxygen saturation in premature babies: a randomized controlled trial. Complementary therapies in clinical practice. 2020;39:101116.

149. Marinelli B, Pluchinotta F, Cozzolino V, Barlafante G, Strozzi MC, Marinelli E, et al. Osteopathic Manipulation Treatment Improves Cerebro–splanchnic Oximetry in Late Preterm Infants. Molecules [Internet]. 4 settembre 2019 [citato 1 aprile 2021];24(18). Disponibile su: https://www.ncbi.nlm.nih.gov/pmc/articles/PMC6767098/

150. Markelz K, Seffinger MA. Spinal manipulation improves pain perception, spinal mobility, and height in men with degenerative disk disease. J Am Osteopath Assoc. 2015;115(1):52–3.

151. Marske C, Bernard N, Palacios A, Wheeler C, Preiss B, Brown M, et al. Fibromyalgia with Gabapentin and Osteopathic Manipulative Medicine: a Pilot Study. Journal of alternative and complementary medicine (New York, NY). 2018;24(4):395-402.

152. Marszalek S, Niebudek-Bogusz E, Woznicka E, Malinska J, Golusinski W, Sliwinska-Kowalska M. Assessment Of the influence of osteopathic myofascial techniques on normalization of the vocal tract functions in patients with occupational dysphonia. Int J Occup Med Environ Health. 2012;25(3):225–35.

153. Marti-Salvador M, Hidalgo-Moreno L, Domenech-Fernandez J, Lison J, Arguisuelas M. Osteopathic Manipulative Treatment Including Specific Diaphragm Techniques Improves Pain and Disability in Chronic Nonspecific Low Back Pain: a Randomized Trial. Archives of physical medicine and rehabilitation. 2018;99(9):1720-1729.

154. Martínez-Ochoa M, Fernández-Domínguez J, Morales-Asencio J, González-Iglesias J, Ricard F, Oliva-Pascual-Vaca Á. Effectiveness of an Osteopathic Abdominal Manual Intervention in Pain Thresholds, Lumbopelvic Mobility, and Posture in Women with Chronic Functional Constipation. Journal of alternative and complementary medicine (New York, NY). 2018;24(8):816-824.

155. Martingano D. Management of Cesarean Deliveries and Cesarean Scars With Osteopathic Manipulative Treatment: A Brief Report. J Am Osteopath Assoc. 1 luglio 2016;116(7):e22-30.

156. Maskey-Warzechowska M, Mierzejewski M, Gorska K, Golowicz R, Jesien L, Krenke R. Effects of Osteopathic Manual Therapy on Hyperinflation in Patients with Chronic Obstructive Pulmonary Disease: A Randomized Cross-Over Study. Adv Exp Med Biol. 2019;1222:17–25.

157. Matsushita S, Wong B, Kanumalla R, Goldstein L. Osteopathic Manipulative Treatment and Psychosocial Management of Dysmenorrhea. J Am Osteopath Assoc. 1 luglio 2020;120(7):479–82.

158. Mazzeo S, Silverberg C, Oommen T, Moya D, Angelo N, Zwibel H, et al. Effects of Osteopathic Manipulative Treatment on Sleep Quality in Student Athletes After Concussion: a Pilot Study. Journal of the American Osteopathic Association [Internet]. 2020; Disponibile su: https://www.cochranelibrary.com/central/doi/10.1002/central/CN-02159406/full

159. McCallister A, Brown C, Smith M, Ettlinger H, Baltazar GA. Osteopathic Manipulative Treatment for Somatic Dysfunction After Acute Severe Traumatic Brain Injury. J Am Osteopath Assoc. 1 dicembre 2016;116(12):810–5.

160. McDermott G, Qureshi Y, Foster-Moumoutjis G, Espejo A. An osteopathic approach to Graves' ophthalmopathy: A case report. International Journal of Osteopathic Medicine. 1 marzo 2020;35:57–61.

161. McPartland JM, Giuffrida A, King J, Skinner E, Scotter J, Musty RE. Cannabimimetic effects of osteopathic manipulative treatment. J Am Osteopath Assoc. giugno 2005;105(6):283–91.

162. McReynolds TM, Sheridan BJ. Intramuscular ketorolac versus osteopathic manipulative treatment in the management of acute neck pain in the emergency department: a randomized clinical trial. J Am Osteopath Assoc. febbraio 2005;105(2):57–68.

163. Melkersson C, Nasic S, Starzmann K, Bengtsson Boström K. Effect of Foot Manipulation on Pregnancy-Related Pelvic Girdle Pain: A Feasibility Study. J Chiropr Med. settembre 2017;16(3):211–9.

164. Ménard M, Ferrari M, Bouchet A, Puchaud P, Vaucher P, Sutre F, et al. Impact of osteopathic manipulative treatment on range of motion of the pelvis during the one-sided tilt test: A pilot study. Comput Methods Biomech Biomed Eng Imaging and Visualization. 2019;22((Ménard M., menard.m@io-rennes.fr; Ferrari M.; Bouchet A.; Sutre F.; Bourgin M.) Institut d'Ostéopathie De Rennes, Bruz, France):S436–8.

165. Mills M, Sevensma K, Serrano J. Osteopathic Manipulative Treatment for a Recognizable Pattern of Somatic Dysfunction Following Laparoscopic Cholecystectomy. J Am Osteopath Assoc. 1 ottobre 2020;120(10):685–90.

166. Mills MV. The use of osteopathic manipulative treatment in the newborn nursery and its effect on health in the first six months of life: A retrospective observational case-control study. Complementary Therapies in Clinical Practice. 1 maggio 2021;43:101357.

167. Mills M, Henley C, Barnes L, Carreiro J, Degenhardt B. The use of osteopathic manipulative treatment as adjuvant therapy in children with recurrent acute otitis media. Archives of pediatrics & adolescent medicine. 2003;157(9):861-866.

168. Mirocha NJ, Parker JD. Successful treatment of refractory functional dyspepsia with osteopathic manipulative treatment. Osteopathic Family Physician. 1 novembre 2012;4(6):193–6.

169. Modlin SE, Borofka K, Franzini D, Klene-Bowns AC, Nuño VA. OMT for the Prevention and Management of Chronic Constipation and Distal Intestinal Obstructive Syndrome in Cystic Fibrosis: A Pilot Study. J Am Osteopath Assoc. 1 luglio 2019;119(7):e31–5.

170. Monaco A, Cozzolino V, Cattaneo R, Cutilli T, Spadaro A. Osteopathic manipulative treatment (OMT) effects on mandibular kinetics: kinesiographic study. Eur J Paediatr Dent. marzo 2008;9(1):37–42.

171. Montrose S, Vogel M, Barber K. Use of osteopathic manipulative treatment for low back pain patients with and without pain medication history. Journal of the American Osteopathic Association. 2021;121(1):63-69.

172. Nemett D, Fivush B, Mathews R, Camirand N, Eldridge M, Finney K, et al. A randomized controlled trial of the effectiveness of osteopathy-based manual physical therapy in treating pediatric dysfunctional voiding. Journal of pediatric urology. 2008;4(2):100-106.

173. Nguyen C, Boutron I, Zegarra-Parodi R, Baron G, Alami S, Sanchez K, et al. Effect of Osteopathic Manipulative Treatment vs Sham Treatment on Activity Limitations in Patients with Nonspecific Subacute and Chronic Low Back Pain: A Randomized Clinical Trial. JAMA Intern Med [Internet]. 2021;((Nguyen C., christelle.nguyen2@aphp.fr; Boutron I.; Daste C.; Lefèvre-Colau M.-M.; Rannou F.) UFR de Médecine, Faculté de Santé, Université de Paris, Paris, France). Disponibile su: https://www.embase.com/search/results?subaction=viewrecord&id=L634485509&from=export

174. Nishida Y, Sopchak MM, Jackson MR, Andersonning TR, Leikert EP, Goldman SI, et al. Effect of select osteopathic manipulative treatment techniques on patients with acute rhinosinusitis. AAO J. 2015;25(3):12–20 and 30.

175. Nobles T, Bach A, Boesler D. Case report of osteopathic treatment of insomnia and traumatic anhidrosis. International Journal of Osteopathic Medicine. 1 settembre 2016;21:58–61.

176. Noccioli G, Noccioli N, Graziosi S, Petracca M, Gambardella O, Dammando F. Randomized clinical trial on the effects of osteopathic treatment in menopausal women. G Ital Ostet Ginecol. 2014;36(2):339–41.

177. Noll DR, Johnson JC, Baer RW, Snider EJ. The immediate effect of individual manipulation techniques on pulmonary function measures in persons with chronic obstructive pulmonary disease. Osteopath Med Prim Care. 8 ottobre 2009;3:9.

178. Noll DR. The short-term effect of a lymphatic pump protocol on blood cell counts in nursing home residents with limited mobility: a pilot study. J Am Osteopath Assoc. luglio 2013;113(7):520–8.

179. Noll DR. The effect of OMT on postoperative medical and functional recovery of coronary artery bypass graft patients. J Am Osteopath Assoc. agosto 2013;113(8):595–6.

180. Noll DR, Degenhardt BF, Johnson JC, Burt SA. Immediate effects of osteopathic manipulative treatment in elderly patients with chronic obstructive pulmonary disease. J Am Osteopath Assoc. maggio 2008;108(5):251–9.

181. Noll DR, Degenhardt BF, Stuart MK, Werden S, McGovern RJ, Johnson JC. The effect of osteopathic manipulative treatment on immune response to the influenza vaccine in nursing homes residents: a pilot study. Altern Ther Health Med. agosto 2004;10(4):74–6.

182. Noll D, Degenhardt B, Stuart M, McGovern R, Matteson M. Effectiveness of a sham protocol and adverse effects in a clinical trial of osteopathic manipulative treatment in nursing home patients. Journal of the American Osteopathic Association. 2004;104(3):107-113.

183. Noll D, Shores J, Bryman P, Masterson E. Adjunctive osteopathic manipulative treatment in the elderly hospitalized with pneumonia: a pilot study. Journal of the American Osteopathic Association. 1999;99(3):143-6, 151-2.

184. Noll D, Shores J, Gamber R, Herron K, Swift J. Benefits of osteopathic manipulative treatment for hospitalized elderly patients with pneumonia. Journal of the American Osteopathic Association. 2000;100(12):776-782.

185. Noll DR; Degenhardt BF; Johnson JC. Multicenter osteopathic pneumonia study in the elderly: subgroup analysis on hospital length of stay, ventilator-dependent respiratory failure rate, and in-hospital mortality rate. The Journal of the American Osteopathic Association 2016 Sep;116(9):574-587. 2016;

186. Nolli L, Baraggiolo S, Pagani M, Origo D, Bergna A, Vismara L. Osteopathic manipulative treatment in soccer players with chronic groin pain: A pilot study. Gazz Med Ital Arch Sci Med. 2017;176(6):322–9.

187. Nuño V, Siu A, Pierce-Talsma S. Osteopathic Manipulative Treatment for Allostatic Load Lowering. J Am Osteopath Assoc. 2019;119(10):e38–9.

188. O’Connor S, Durand M-J, Hudson M, Baron M, Gaudreault N. Effects of osteopathic manipulative treatment on hand function, disease symptoms and functional status in systemic sclerosis: a series of single-case studies in working women. International Journal of Osteopathic Medicine. 1 dicembre 2016;22:21–32.

189. Origo D, Tarantino AG. Osteopathic manipulative treatment in pudendal neuralgia: A case report. J Bodyw Mov Ther. aprile 2019;23(2):247–50.

190. Origo D, Tarantino AG, Nonis A, Vismara L. Osteopathic manipulative treatment in chronic coccydynia: A case series. J Bodyw Mov Ther. aprile 2018;22(2):261–5.

191. Origo D, Tarantino AG, Romagnoli M. Vestibular failure managed with osteopathic manipulative treatment: A report of two cases. J Bodyw Mov Ther. luglio 2020;24(3):59–62.

192. Papa L, Amodio A, Biffi F, Mandara A. Impact of osteopathic therapy on proprioceptive balance and quality of life in patients with dizziness. J Bodyw Mov Ther. ottobre 2017;21(4):866–72.

193. Papa L, Mandara A, Bottali M, Gulisano V, Orfei S. A randomized control trial on the effectiveness of osteopathic manipulative treatment in reducing pain and improving the quality of life in elderly patients affected by osteoporosis. Clin Cases Miner Bone Metab. 2012;9(3):179–83.

194. Parker J, Heinking KP, Kappler RE. Efficacy of osteopathic manipulative treatment for low back pain in euhydrated and hypohydrated conditions: a randomized crossover trial. J Am Osteopath Assoc. maggio 2012;112(5):276–84.

195. Paul L, Berkowitz MR. Osteopathic manipulative treatment for an unusual presentation of fibromyalgia: A case report demonstrating the effectiveness of disease guidelines. International Journal of Osteopathic Medicine. 1 giugno 2015;18(2):141–7.

196. Pellerin F, Papin-Richard E, Guihéneuc P, Niel S, Guihard G. Can osteopathic manipulative treatment modify the posture in elderly people? – A single-case study. Journal of Bodywork and Movement Therapies. 1 aprile 2015;19(2):380–8.

197. Philippi H, Faldum A, Schleupen A, Pabst B, Jung T, Bergmann H, et al. Infantile postural asymmetry and osteopathic treatment: a randomized therapeutic trial. Dev Med Child Neurol. gennaio 2006;48(1):5–9; discussion 4.

198. Piche T, Pishvaie D, Tirouvaziam D, Filippi J, Dainese R, Tonohouhan M, et al. Osteopathy decreases the severity of IBS-like symptoms associated with Crohn's disease in patients in remission. European journal of gastroenterology & hepatology. 2014;26(12):1392-1398.

199. Pizzolorusso G, Cerritelli F, Accorsi A, Lucci C, Tubaldi L, Lancellotti J, et al. The effect of optimally timed osteopathic manipulative treatment on length of hospital stay in moderate and late preterm infants: results from a RCT. Evidence-based complementary and alternative medicine [Internet]. 2014;2014. Disponibile su: https://www.cochranelibrary.com/central/doi/10.1002/central/CN-01037198/full

200. Pizzolorusso G, Turi P, Barlafante G, Cerritelli F, Renzetti C, Cozzolino V, et al. Effect of osteopathic manipulative treatment on gastrointestinal function and length of stay of preterm infants: an exploratory study. Chiropractic & manual therapies [Internet]. 2011;19. Disponibile su: https://www.cochranelibrary.com/central/doi/10.1002/central/CN-00900552/full

201. Plotkin BJ, Rodos JJ, Kappler R, Schrage M, Freydl K, Hasegawa S, et al. Adjunctive osteopathic manipulative treatment in women with depression: a pilot study. J Am Osteopath Assoc. settembre 2001;101(9):517–23.

202. Ponzo V, Cinnera AM, Mommo F, Caltagirone C, Koch G, Tramontano M. Osteopathic manipulative therapy potentiates motor cortical plasticity. J Am Osteopath Assoc. 2018;118(6):396–402.

203. Porcari B, Russo M, Naro A, La Via C, Pullia M, Accorinti M, et al. Effects of osteopathic manipulative treatment on patients with multiple sclerosis: A pilot study. Complement Ther Med. aprile 2019;43:154–6.

204. Probst P, Buchler E, Doerr-Harim C, Knebel P, Thiel B, Ulrich A, et al. Randomised controlled pilot trial on feasibility, safety and effectiveness of osteopathic MANipulative treatment following major abdominal surgery (OMANT pilot trial). International journal of osteopathic medicine. 2016;20:31-40.

205. Racca V, Bordoni B, Castiglioni P, Modica M, Ferratini M. Osteopathic Manipulative Treatment Improves Heart Surgery Outcomes: A Randomized Controlled Trial. Ann Thorac Surg. luglio 2017;104(1):145–52.

206. Rajaii RM, Cox GJ, Schneider RP. Role of osteopathic manipulative treatment in the management of stiff person syndrome. J Am Osteopath Assoc. giugno 2015;115(6):394–8.

207. Ridgeway V, Berkowitz MR. Somatic dysfunction following sigmoid colon resection for diverticulitis: A case report. AAO J. 2010;20(2):25-26+28.

208. Rolle G, Tremolizzo L, Somalvico F, Ferrarese C, Bressan L. Pilot trial of osteopathic manipulative therapy for patients with frequent episodic tension-type headache. Journal of the American Osteopathic Association. 2014;114(9):678-685.

209. Roncada G. Effects of osteopathic treatment on pulmonary function and chronic thoracic pain after coronary artery bypass graft surgery (OstinCaRe): study protocol for a randomised controlled trial. BMC complementary and alternative medicine. 2016;16(1):482.

210. Roncada G. Osteopathic treatment leads to significantly greater reductions in chronic thoracic pain after CABG surgery: a randomised controlled trial. Journal of bodywork and movement therapies. 2020;24(3):202-211.

211. Ross BS, Johnson VM. Osteopathic manipulative treatment in vestibular neuritis: A case report. AAO J. 2014;24(2):27-30+32.

212. Ross G, Macfarlane C, Vaughan B. Combined osteopathy and exercise management of Achilles tendinopathy in an athlete. J Sports Med Phys Fitness. febbraio 2018;58(1–2):106–12.

213. Rotter G, Fernholz I, Binting S, Keller T, Roll S, Kass B, et al. The effect of osteopathic medicine on pain in musicians with nonspecific chronic neck pain: a randomized controlled trial. Therapeutic advances in musculoskeletal disease [Internet]. 2020;12. Disponibile su: https://www.cochranelibrary.com/central/doi/10.1002/central/CN-02213960/full

214. Ruffini N, D'Alessandro G, Mariani N, Pollastrelli A, Cardinali L, Cerritelli F. Variations of high frequency parameter of heart rate variability following osteopathic manipulative treatment in healthy subjects compared to control group and sham therapy: randomized controlled trial. Front Neurosci [Internet]. 4 agosto 2015 [citato 1 aprile 2021];9. Disponibile su: https://www.ncbi.nlm.nih.gov/pmc/articles/PMC4523739/

215. Sandhouse ME, Shechtman D, Sorkin R, Drowos JL, Caban-Martinez AJ, Patterson MM, et al. Effect of osteopathy in the cranial field on visual function-a pilot study. J Am Osteopath Assoc. 2010;110(4):239–43.

216. Sandhouse ME, Shechtman D, Fecho G, Timoshkin EM. Effect of Osteopathic Cranial Manipulative Medicine on Visual Function. J Am Osteopath Assoc. 1 novembre 2016;116(11):706–14.

217. Schwerla F, Bischoff A, Nurnberger A, Genter P, Guillaume J, Resch K. Osteopathic treatment of patients with chronic non-specific neck pain: a randomised controlled trial of efficacy. Forschende Komplementarmedizin (2006). 2008;15(3):138-145.

218. Schwerla F, Hinse T, Klosterkamp M, Schmitt T, Rutz M, Resch K-L. Osteopathic treatment of patients with shoulder pain. A pragmatic randomized controlled trial. Journal of bodywork and movement therapies. 2020;24(3):21-28.

219. Schwerla F, Rother K, Rother D, Ruetz M, Resch K. Osteopathic Manipulative Therapy in Women With Postpartum Low Back Pain and Disability: a Pragmatic Randomized Controlled Trial. Journal of the American Osteopathic Association. 2015;115(7):416-425.

220. Schwerla F, Kaiser AK, Gietz R, Kastner R. Osteopathic treatment of patients with long-term sequelae of whiplash injury: effect on neck pain disability and quality of life. J Altern Complement Med. giugno 2013;19(6):543–9.

221. Schwerla F, Wirthwein P, Rütz M, Resch K-L. Osteopathic treatment in patients with primary dysmenorrhoea: A randomised controlled trial. International Journal of Osteopathic Medicine. 1 dicembre 2014;17(4):222–31.

222. Scoppa, Pirino A, Belloni G, Gallamini M, Messina G, Iovane A. Postural and autonomic modifications following osteopathic manipulative treatment (OMT): Comparison between two techniques. A pilot study. Acta Med Mediterr. 2018;34(2):431–6.

223. Seidel B, Desipio GB. Use of osteopathic manipulative treatment to manage recurrent bouts of singultus. J Am Osteopath Assoc. agosto 2014;114(8):660–4.

224. Seiler M, Vermeylen B, Poortmans B, Feipel V, Dugailly P-M. Effects of non-manipulative osteopathic management in addition to physical therapy and rehabilitation on clinical outcomes of ankylosing spondylitis patients: A preliminary randomized clinical trial. Journal of Bodywork and Movement Therapies. ottobre 2020;24(4):51–6.

225. Shadiack E 3rd, Jouett N, van den Raadt A, Liganor R, Watters J, Hensel K, et al. Osteopathic Manipulative Treatment Alters Gastric Myoelectric Activity in Healthy Subjects. J Altern Complement Med. dicembre 2018;24(12):1176–80.

226. Shah R, Berkowitz MR. Osteopathic manipulative treatment of isolated chronic sphenoidal sinusitis in a post-sinus surgery patient: A case report. AAO J. 2011;21(1):24–7.

227. Shanahan LKT, Raines SGM, Coggins RL, Moore T, Carnes M, Griffin L. Osteopathic Manipulative Treatment in the Management of Isaacs Syndrome. J Am Osteopath Assoc. 1 marzo 2017;117(3):194–8.

228. Sillem M, Juhasz-Böss I, Klausmeier I, Mechsner S, Siedentopf F, Solomayer E. Osteopathy for Endometriosis and Chronic Pelvic Pain – a Pilot Study. Geburtshilfe Frauenheilkd. settembre 2016;76(9):960–3.

229. Silva A, Biasotto-Gonzalez D, Oliveira F, Andrade A, Gomes C, Lanza F, et al. Effect of Osteopathic Visceral Manipulation on Pain, Cervical Range of Motion, and Upper Trapezius Muscle Activity in Patients with Chronic Nonspecific Neck Pain and Functional Dyspepsia: a Randomized, Double-Blind, Placebo-Controlled Pilot Study. Evidence-based complementary and alternative medicine. 2018;2018(no pagination):4929271.

230. Siu G, Desai A, Manne BB, Mason D, Weinik MM. Rehabilitation and osteopathic manipulative medicine for a patient with dysphagia secondary to a hyoid somatic dysfunction: A case report. PM R. 2010;2(9):S91–2.

231. Smallwood CR, Borgerding CJ, Cox MS, Berkowitz MR. Osteopathic manipulative treatment (OMT) during labor facilitates a natural, drug-free childbirth for a primigravida patient: A case report. International Journal of Osteopathic Medicine. settembre 2013;16(3):170–7.

232. Smilowicz A. An osteopathic approach to gastrointestinal disease: somatic clues for diagnosis and clinical challenges associated with Helicobacter pylori antibiotic resistance. J Am Osteopath Assoc. maggio 2013;113(5):404–16.

233. Smith L, Berkowitz MR. Osteopathic approach to chronic constipation in Prader–Willi Syndrome: A case report. International Journal of Osteopathic Medicine. 1 marzo 2016;19:73–7.

234. Snider. Ultrasonographic Evaluation of the Effect of Osteopathic Manipulative Treatment on Sacral Base Asymmetry. Journal of the American Osteopathic Association. 2018;118(3):159-169.

235. Snider KT. The use of osteopathic manipulative treatment as part of an integrated treatment for infantile colic:A case report. AAO J. 2016;26(2):15–8 and 33.

236. Snider KT. The use of osteopathic manipulative treatment for acute dental Pain: A case report. AAO J. 2016;26(1):17–24.

237. Snider K, Snider E, Johnson J, Hagan C, Schoenwald C. Preventative osteopathic manipulative treatment and the elderly nursing home resident: a pilot study. Journal of the American Osteopathic Association. 2012;112(8):489-501.

238. Snyder LL, Knox SC, Smutny CJ. Integrating Osteopathic Manipulative Treatment and Injections in the Diagnosis and Management of a Hip Labral Tear. J Am Osteopath Assoc. 1 giugno 2020;120(6):421–4.

239. Sonberg M, Mullinger B, Rajendran D. Can osteopathy help women with a history of hypothyroidism and musculoskeletal complaints? Outcome of a preliminary, prospective, open investigation. International Journal of Osteopathic Medicine. 1 marzo 2010;13(1):11–6.

240. Steele KM, Viola J, Burns E, Carreiro JE. Brief report of a clinical trial on the duration of middle ear effusion in young children using a standardized osteopathic manipulative medicine Protocol. J Am Osteopath Assoc. 2010;110(5):278–84.

241. Steele KM, Carreiro JE, Viola JH, Conte JA, Ridpath LC. Effect of osteopathic manipulative treatment on middle ear effusion following acute otitis media in young children: a pilot study. J Am Osteopath Assoc. giugno 2014;114(6):436–47.

242. Stępnik J, Kędra A, Czaprowski D. Short-term effect of osteopathic manual techniques (OMT) on respiratory function in healthy individuals. PLoS One. 2020;15(6):e0235308.

243. Sucher BM. Ultrasonography-guided osteopathic manipulative treatment for a patient with thoracic outlet syndrome. J Am Osteopath Assoc. 2011;111(9):543–7.

244. Summers GK, Lewis DD. An osteopathic approach to low back pain and short leg syndrome in a patient with traumatic brain injury following motor vehicle crash: A case report. AAO J. 2018;28(3):12–7.

245. Swender D, Thompson G, Schneider K, McCoy K, Patel A. Osteopathic manipulative treatment for inpatients with pulmonary exacerbations of cystic fibrosis: effects on spirometry findings and patient assessments of breathing, anxiety, and pain. Journal of the American Osteopathic Association. 2014;114(6):450-458.

246. Tamburella F, Piras F, Piras F, Spanò B, Tramontano M, Gili T. Cerebral Perfusion Changes After Osteopathic Manipulative Treatment: A Randomized Manual Placebo-Controlled Trial. Front Physiol. 2019;10:403.

247. Tamer S, Öz M, Ülger Ö. The effect of visceral osteopathic manual therapy applications on pain, quality of life and function in patients with chronic nonspecific low back pain. J Back Musculoskelet Rehabil. 2017;30(3):419–25.

248. Tarsuslu T, Bol H, Simşek IE, Toylan IE, Cam S. The effects of osteopathic treatment on constipation in children with cerebral palsy: a pilot study. J Manipulative Physiol Ther. ottobre 2009;32(8):648–53.

249. Thomaz S, Teixeira F, de Lima A, Cipriano Júnior G, Formiga M, Cahalin L. Osteopathic manual therapy in heart failure patients: a randomized clinical trial. Journal of bodywork and movement therapies. 2018;22(2):293-299.

250. Tramontano M, Pagnotta S, Lunghi C, Manzo C, Manzo F, Consolo S, et al. Assessment and Management of Somatic Dysfunctions in Patients With Patellofemoral Pain Syndrome. J Am Osteopath Assoc. 1 marzo 2020;120(3):165–73.

251. Urbanek B. Osteopathic care of a twin pregnancy - A case example. Osteopath Med. 2015;16(1):16–8.

252. Van Attali T, Bouchoucha M, Benamouzig R. Randomised Prospective Evaluation of Osteopathic Treatment for Irritable Bowel Syndrome. Gastroenterology. 1 maggio 2011;140(5, Supplement 1):S-614.

253. Van Ravenswaay VJ, Hain SJ, Grasso S, Shubrook JH. Effects of Osteopathic Manipulative Treatment on Diabetic Gastroparesis. J Am Osteopath Assoc. luglio 2015;115(7):452–8.

254. Vandenplas Y, Denayer E, Vandenbossche T, Vermet L, Hauser B, Deschepper J, et al. Osteopathy may decrease obstructive apnea in infants: a pilot study. Osteopath Med Prim Care. 19 luglio 2008;2:8.

255. Villalta Santos L, Lisboa Córdoba L, Benite Palma Lopes J, Santos Oliveira C, André Collange Grecco L, Bovi Nunes Andrade AC, et al. Active Visceral Manipulation Associated With Conventional Physiotherapy in People With Chronic Low Back Pain and Visceral Dysfunction: A Preliminary, Randomized, Controlled, Double-Blind Clinical Trial. J Chiropr Med. giugno 2019;18(2):79–89.

256. Vismara L, Cimolin V, Menegoni F, Zaina F, Galli M, Negrini S, et al. Osteopathic manipulative treatment in obese patients with chronic low back pain: a pilot study. Manual therapy. 2012;17(5):451-455.

257. Vismara L, Cimolin V, Galli M, Grugni G, Ancillao A, Capodaglio P. Osteopathic Manipulative Treatment improves gait pattern and posture in adult patients with Prader–Willi syndrome. International Journal of Osteopathic Medicine. 1 marzo 2016;19:35–43.

258. Vismara L, Cozzolino V, Pradotto LG, Gentile R, Tarantino AG. Severe Postoperative Chronic Constipation Related to Anorectal Malformation Managed with Osteopathic Manipulative Treatment. Case Rep Gastroenterol. aprile 2020;14(1):220–5.

259. Voigt K, Liebnitzky J, Burmeister U, Sihvonen-Riemenschneider H, Beck M, Voigt R, et al. Efficacy of osteopathic manipulative treatment of female patients with migraine: results of a randomized controlled trial. J Altern Complement Med. marzo 2011;17(3):225–30.

260. Wahl RA, Aldous MB, Worden KA, Grant KL. Echinacea purpurea and osteopathic manipulative treatment in children with recurrent otitis media: a randomized controlled trial. BMC Complement Altern Med. 2 ottobre 2008;8:56.

261. Walkowski S, Singh M, Puertas J, Pate M, Goodrum K, Benencia F. Osteopathic manipulative therapy induces early plasma cytokine release and mobilization of a population of blood dendritic cells. PLoS ONE [Internet]. 2014;9(3). Disponibile su: https://www.embase.com/search/results?subaction=viewrecord&id=L372749809&from=export

262. Wells MR, Giantinoto S, D'Agate D, Areman RD, Fazzini EA, Dowling D, et al. Standard osteopathic manipulative treatment acutely improves gait performance in patients with Parkinson's disease. J Am Osteopath Assoc. 1999;99(2):92–8.

263. Wetzler G, Roland M, Fryer-Dietz S, Dettmann-Ahern D. CranioSacral Therapy and Visceral Manipulation: A New Treatment Intervention for Concussion Recovery. Med Acupunct. 1 agosto 2017;29(4):239–48.

264. Wiegand S, Bianchi W, Quinn T, Best M, Fotopoulos T. Osteopathic manipulative treatment for self-reported fatigue, stress, and depression in first-year osteopathic medical students. Journal of the American Osteopathic Association. 2015;115(2):84-93.

265. Wieting J, Beal C, Roth G, Gorbis S, Dillard L, Gilliland D, et al. The effect of osteopathic manipulative treatment on postoperative medical and functional recovery of coronary artery bypass graft patients. Journal of the American Osteopathic Association. 2013;113(5):384-393.

266. Williams NH, Wilkinson C, Russell I, Edwards RT, Hibbs R, Linck P, et al. Randomized osteopathic manipulation study (ROMANS): pragmatic trial for spinal pain in primary care. Fam Pract. dicembre 2003;20(6):662–9.

267. Winter J, Kimber A, Montenegro S, Gao J. Ultrasonography to Assess the Efficacy of Osteopathic Manipulative Treatment for Lumbar Spine Asymmetry. J Am Osteopath Assoc. 2020;120(11):761–9.

268. Wójcik M, Dziembowska I, Izdebski P, Żekanowska E. Pilot randomized single-blind clinical trial, craniosacral therapy vs control on physiological reaction to math task in male athletes. International Journal of Osteopathic Medicine. 1 giugno 2019;32:7–12.

269. Wyatt K, Edwards V, Franck L, Britten N, Creanor S, Maddick A, et al. Cranial osteopathy for children with cerebral palsy: A randomised controlled trial. Arch Dis Child. 2011;96(6):505–12.

270. Yao S, Zwibel H, Angelo N, Leder A, Mancini J. Effectiveness of Osteopathic Manipulative Medicine vs Concussion Education in Treating Student Athletes With Acute Concussion Symptoms. Journal of the American Osteopathic Association [Internet]. 2020; Disponibile su: https://www.cochranelibrary.com/central/doi/10.1002/central/CN-02159535/full

271. Yates HA, Vardy TC, Kuchera ML, Ripley BD, Johnson JC. Effects of osteopathic manipulative treatment and concentric and eccentric maximal-effort exercise on women with multiple sclerosis: a pilot study. J Am Osteopath Assoc. maggio 2002;102(5):267–75.

272. Younes M, Nowakowski K, Didier-Laurent B, Gombert M, Cottin F. Effect of spinal manipulative treatment on cardiovascular autonomic control in patients with acute low back pain. Chiropractic & manual therapies. 2017;25:33.

273. Zago J, Amatuzzi F, Rondinel T, Matheus J. Osteopathic Manipulative Treatment Versus Exercise Program in Runners With Patellofemoral Pain Syndrome: a Randomized Controlled Trial. Journal of sport rehabilitation. 2020;1-10.

274. Zaidi T, Williams S. The use of OMT in patients with osteoarthritis: Case report. AAO J. 2006;16(4):25–7.

275. Zanotti E, Berardinelli P, Bizzarri C, Civardi A, Manstretta A, Rossetti S, et al. Osteopathic manipulative treatment effectiveness in severe chronic obstructive pulmonary disease: a pilot study. Complement Ther Med. aprile 2012;20(1–2):16–22.

276. Zarucchi A, Vismara L, Frazzitta G, Mauro A, Priano L, Maestri R, et al. Efficacy of Osteopathic Manipulative Treatment on postural control in Parkinsonian patients with Pisa syndrome: A pilot randomized placebo-controlled trial. NeuroRehabilitation. 2020;46(4):529–37.

277. Zecchillo D, Acquati A, Aquino A, Pisa V, Uberti S, Ratti S. Osteopathic Manipulative Treatment of Primary Dysmenorrhea and Related Factors: A Randomized Controlled Trial. International journal of health sciences. 11 dicembre 2017;6:165–74.

Table S3. **Preferred Reporting Items for Systematic reviews and Meta-Analyses extension for Scoping Reviews (PRISMA-ScR) Checklist**

SECTION	ITEM	PRISMA-ScR CHECKLIST ITEM	REPORTED ON PAGE #
**TITLE**			
Title	1	Identify the report as a scoping review.	1
**ABSTRACT**			
Structured summary	2	Provide a structured summary that includes (as applicable): background, objectives, eligibility criteria, sources of evidence, charting methods, results, and conclusions that relate to the review questions and objectives.	1
**INTRODUCTION**			
Rationale	3	Describe the rationale for the review in the context of what is already known. Explain why the review questions/objectives lend themselves to a scoping review approach.	1,2
Objectives	4	Provide an explicit statement of the questions and objectives being addressed with reference to their key elements (e.g., population or participants, concepts, and context) or other relevant key elements used to conceptualize the review questions and/or objectives.	2
**METHODS**			
Protocol and registration	5	Indicate whether a review protocol exists; state if and where it can be accessed (e.g., a Web address); and if available, provide registration information, including the registration number.	2
Eligibility criteria	6	Specify characteristics of the sources of evidence used as eligibility criteria (e.g., years considered, language, and publication status), and provide a rationale.	3
Information sources*	7	Describe all information sources in the search (e.g., databases with dates of coverage and contact with authors to identify additional sources), as well as the date the most recent search was executed.	3

SECTION	ITEM	PRISMA-ScR CHECKLIST ITEM	REPORTED ON PAGE #
Search	8	Present the full electronic search strategy for at least 1 database, including any limits used, such that it could be repeated.	3
Selection of sources of evidence†	9	State the process for selecting sources of evidence (i.e., screening and eligibility) included in the scoping review.	3
Data charting process‡	10	Describe the methods of charting data from the included sources of evidence (e.g., calibrated forms or forms that have been tested by the team before their use, and whether data charting was done independently or in duplicate) and any processes for obtaining and confirming data from investigators.	4
Data items	11	List and define all variables for which data were sought and any assumptions and simplifications made.	4
Critical appraisal of individual sources of evidence§	12	If done, provide a rationale for conducting a critical appraisal of included sources of evidence; describe the methods used and how this information was used in any data synthesis (if appropriate).	4
Synthesis of results	13	Describe the methods of handling and summarizing the data that were charted.	4
**RESULTS**			
Selection of sources of evidence	14	Give numbers of sources of evidence screened, assessed for eligibility, and included in the review, with reasons for exclusions at each stage, ideally using a flow diagram.	4-8
Characteristics of sources of evidence	15	For each source of evidence, present characteristics for which data were charted and provide the citations.	5-8
Critical appraisal within sources of evidence	16	If done, present data on critical appraisal of included sources of evidence (see item 12).	5-8
Results of individual sources of evidence	17	For each included source of evidence, present the relevant data that were charted that relate to the review questions and objectives.	5-8
Synthesis of results	18	Summarize and/or present the charting results as they relate to the review questions and objectives.	5-8
**DISCUSSION**			
Summary of evidence	19	Summarize the main results (including an overview of concepts, themes, and types of evidence available), link to the review questions and objectives, and consider the relevance to key groups.	9-11
Limitations	20	Discuss the limitations of the scoping review process.	11
Conclusions	21	Provide a general interpretation of the results with respect to the review questions and objectives, as well as potential implications and/or next steps.	12
**FUNDING**			
Funding	22	Describe sources of funding for the included sources of evidence, as well as sources of funding for the scoping review. Describe the role of the funders of the scoping review.	12

JBI = Joanna Briggs Institute; PRISMA-ScR = Preferred Reporting Items for Systematic reviews and Meta-Analyses extension for Scoping Reviews.

* Where *sources of evidence* (see second footnote) are compiled from, such as bibliographic databases, social media platforms, and Web sites.

† A more inclusive/heterogeneous term used to account for the different types of evidence or data sources (e.g., quantitative and/or qualitative research, expert opinion, and policy documents) that may be eligible in a scoping review as opposed to only studies. This is not to be confused with *information sources* (see first footnote).

‡ The frameworks by Arksey and O'Malley (6) and Levac and colleagues (7) and the JBI guidance (4, 5) refer to the process of data extraction in a scoping review as data charting.

§ The process of systematically examining research evidence to assess its validity, results, and relevance before using it to inform a decision. This term is used for items 12 and 19 instead of "risk of bias" (which is more applicable to systematic reviews of interventions) to include and acknowledge the various sources of evidence that may be used in a scoping review (e.g., quantitative and/or qualitative research, expert opinion, and policy document).

From: Tricco AC, Lillie E, Zarin W, O'Brien KK, Colquhoun H, Levac D, et al. PRISMA Extension for Scoping Reviews (PRISMAScR): Checklist and Explanation. Ann Intern Med. 2018;169:467–473. doi: 10.7326/M18-0850.
